# Recent advancements and future prospects on AI-integrated sensing techniques for non-invasive chronic kidney disease diagnosis: a review

**DOI:** 10.3389/frai.2026.1836646

**Published:** 2026-08-13

**Authors:** Suchetha Manikandan, Preethi Senthilkumar, Shivani Selvachandran, Jim Elliot Christopherjames, Shabbir Syed Abdul

**Affiliations:** 1 Centre for Healthcare Advancement, Innovation and Research, Vellore Institute of Technology, Chennai, India; 2 Malla Reddy Institute of Medical Sciences, Hyderabad, India; 3 Graduate Institute of Biomedical Informatics, Taipei Medical University, Taipei, Taiwan

**Keywords:** breath, Chronic Kidney Disease, non-invasive diagnosis, saliva, sweat

## Abstract

Chronic Kidney Disease (CKD) has emerged as a major public health concern worldwide, and most patients with CKD are asymptomatic until the later stages, causing growing morbidity and mortality. Diabetes and hypertension are the main causative factors for the development of CKD, damaging the renal microcirculation system. In addition, the impact of Acute Kidney Injuries (AKI) may result in the recovery or progression to either CKD or renal failure. The conventional techniques for diagnosis, such as the measurement of blood creatinine levels, are invasive and time-consuming and may also overlook the early stages of CKD. The non-invasive technology for the diagnosis of CKD has experienced tremendous improvements with the development in the field of sensing technology and the revolution in the field of Artificial Intelligence. This review article covers the non-invasive sensing technology using the non-invasive biofluids/biological matrices, such as saliva, breath, and sweat, for the diagnosis of Chronic Kidney Disease. Additionally, the framework for incorporating Machine Learning models for the automated prediction of CKD in its early stages is analysed.

## 1 Introduction

Chronic Kidney Disease is a condition that develops slowly, leading to the continuous reduction of kidney performance. Nearly 800 million adults live with CKD as per latest global estimates (Hill et al., 2016). The global prevalence of CKD is estimated at 10.4% among men and 11.8% among women. By 2030, the diagnostic market for CKD is estimated to increase to $1.39 billion. The market value is around $850 million by the year 2022 and it has increases substantially (Liu et al., 2024). CKD afflicts around 10% of the world population, and millions of people die every year (Levey et al., 2005, 2020; Cedillo-Flores et al., 2025). Kidney disease refers to the inability of the kidneys to perform their normal filtering functions. As the severity of CKD increases, waste products accumulate dangerously within the body (El Nahas and Bello, 2005; Nimmagadda et al., 2023). Each year, millions of people lose their lives to this condition. Recent medical reports indicate that around 323 million people globally are impacted by CKD. Due to the challenges in forecasting the decline of kidney function, consistent monitoring is crucial. CKD leads to cardiovascular issues, such as heart attacks, hypertension, strokes, etc. The kidneys are responsible for removing waste products, surplus fluids, and toxins, and to regulate blood pressure, to maintain biochemical elements. When the kidney is not able to filter the biochemical substances, blood elements and proteins can seep into saliva, sweat, breath, urine in different proportions of trace elements. Kidney diseases are more prevalent among older adults. If left untreated, CKD can progress to complete kidney failure. In such cases, survival depends on dialysis or kidney transplantation to replace lost function (Levey et al., 2007; Nimmagadda et al., 2023).

CKD affects patients between the age group of 30-50 years. High blood pressure or high blood sugar can be the possible causes for patients with CKD and may result in various complications. CKD is a significant healthcare problem across the world. It is a clinically underrated condition. Today, it is well recognized that CKD is a silent condition where very often the presenting symptoms occur after considerable renal damage has occurred. Conventionally, diagnostic and staging work has focused on serum creatinine levels and the urine albumin-to-creatinine ratio (uACR). These have been well-validated techniques but involve obtaining body fluids. Such techniques can be prone to individual variation and may not be sensitive enough for picking up changes before the renal impairment has become overt. A substantial need in the scientific literature for the development of new methodologies that would allow for earlier, easier, and minimally invasive diagnosis of CKD. In recent years, there have been substantial developments in biosensory technology, breathomics, and machine learning in the field of nephrology; however, there currently appears to be a lack of overlap in the literature between all three of these areas in developing a functional diagnosis platform. Although it appears that there are volatile markers of ammonia in the breath of those individuals with CKD, there does not seem to be extensive development of breath diagnosis in this area.

### 1.1 Stages of chronic kidney disease

**Figure 1:**
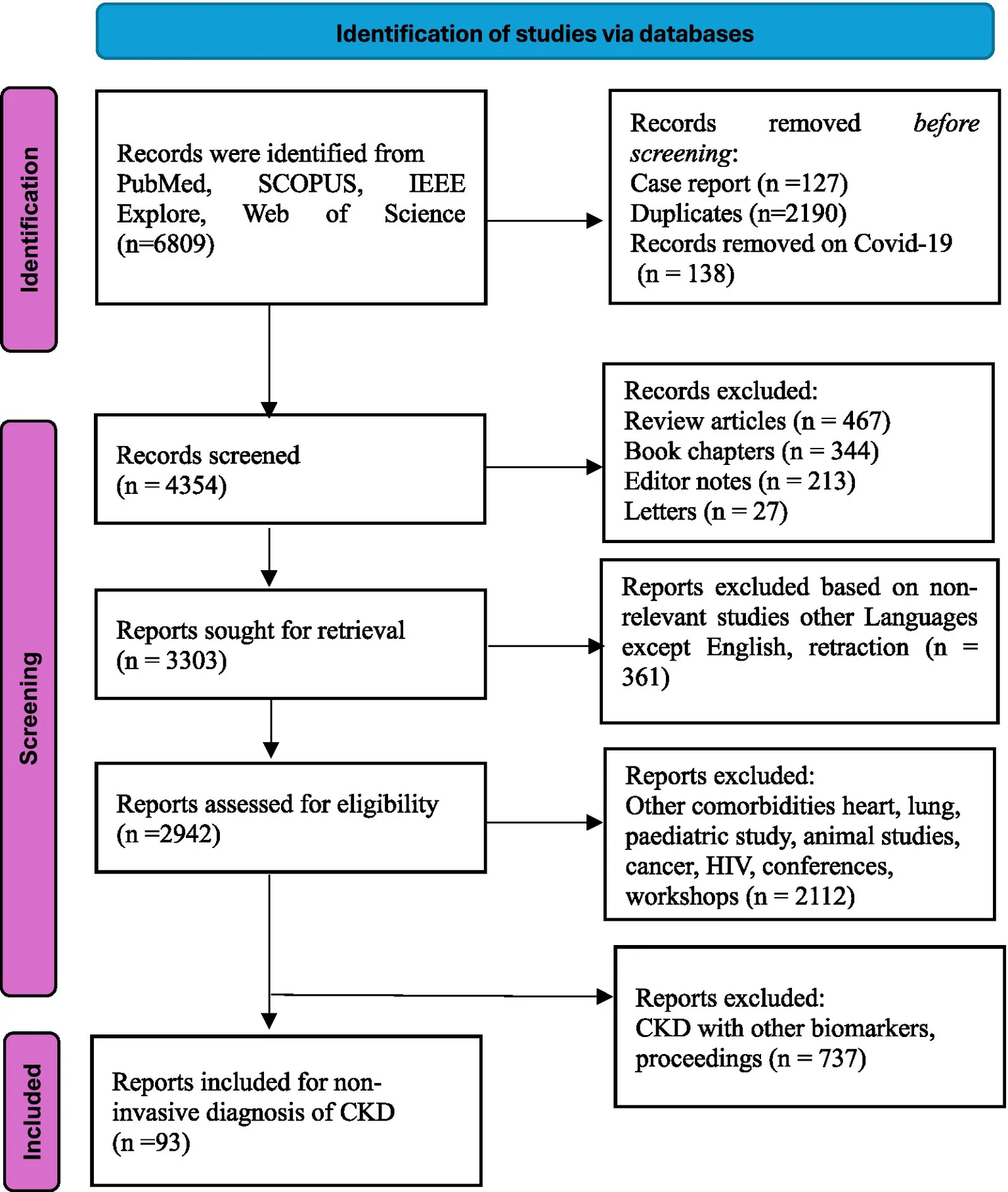
PRISMA flow diagram demonstrating the study selection.

CKD is classified into five distinct stages according to the progressive decline in renal filtration capacity. This functional impairment is quantitatively assessed using the glomerular filtration rate (GFR), a standardized indicator of kidney performance (Schwartz and Furth, 2007). The corresponding GFR thresholds defining each stage as shown in Table 1 of CKD are illustrated in Figure 1. Stage 1 and 2 of CKD corresponds to mild damage to the kidneys. These two stages of kidney disease usually do not show any symptoms. GFR value between 30 and 59 means that the kidneys are moderately affected, and they are not functioning as they should. The GFR value in the range of 15 to 29 indicates that the kidneys are severely damaged. People with CKD stage 1-3 can be treated so that the disease does not advance to severe stages. Stage 4 renal disorder must be taken very seriously as this is the last stage before renal failure (Nimmagadda et al., 2023). The final stage of CKD corresponds to the complete failure of the kidneys.

**Table 1:** CKD stages and the condition of the kidney with their GFR values.

Stages	GFR (ml/min/1.73m^2^)	Condition of the kidney
1	*>*90	Slight damage
2	60-89	Mild decrease in function
3	30 -59	Moderately affected
4	15-29	Seriously damaged
5	*<*15	Completely failed

At this stage, the kidneys will not be able to remove the waste products from the body. This will lead to the buildup of toxins in the blood, which will create severe health problems for the patient. The good thing about CKD is that kidney damage does not happen suddenly. As the kidney disease usually advances gradually over a period of years, early detection can help to slow down further damage to the kidneys (Schwartz and Furth, 2007; Levey et al., 2011; Nimmagadda et al., 2023).

## 2 Methodology

A systematic literature review was conducted following the PRISMA (Preferred Reporting Items for Systematic Reviews and Meta-Analyses) 2020 guidelines (Haddaway et al., 2022) to explore research concerning artificial intelligence (AI) enabled non-invasive biomarkers and biosensors for diagnosing and monitoring CKD. A complete search was carried out across four electronic databases such as PubMed, Scopus, Web of Science, and IEEE Xplore, from January 2000 to March 2026. The search strategy involves the following keywords like chronic kidney disease, CKD, renal disease, saliva, sweat, breath, exhaled breath, biosensor, wearable sensor, electrochemical sensor, machine learning, artificial intelligence, deep learning, XAI, creatinine, urea, eGFR, albuminuria, proteinuria using Boolean operators. Only peer-reviewed journal research articles were included, while editorials, review articles, letters, book chapters, case reports, theses, patents, conference papers, proceedings, and publications in languages other than English were excluded. Initially, 6,809 records were identified, from which 2,190 duplicates were removed along with case reports and articles related to COVID-19, leaving 4,354 records for screening. After reviewing titles and abstracts, 1,051 records were excluded, and 127 reports were inaccessible. Additional exclusions comprised 2,112 conference papers, 13 retracted articles, and 347 non-english publications, among others. After thorough assessment based on predefined inclusion and exclusion criteria, 93 studies were selected for qualitative synthesis to be included in the article. These studies were chosen for their focus on AI-driven analysis of non-invasive biological samples and their potential application in the early diagnosis of CKD.

## 3 Review on CKD biomarkers

Serum urea and creatinine serve as the most widely used and clinically validated biomarkers for CKD detection (Fassett et al., 2011). Beyond maintaining fluid balance, kidneys primarily filter waste products such as creatinine and urea from circulation (El Nahas and Bello, 2005). Blood tests routinely measure serum levels of these markers to assess renal function (Stevens and Levey, 2009). Elevated creatinine and urea concentrations inversely correlate with glomerular filtration capacity (Fassett et al., 2011). Creatinine, a byproduct of constant muscle metabolism, exhibits stable daily production in healthy individuals before renal filtration and urinary excretion (Stevens and Levey, 2009). In a similar manner, urea is produced through the hepatic conversion of ammonia derived from proteins, which is a toxic intermediate, and is subsequently eliminated as a non-toxic waste product by the kidneys (Renda, 2017). Consistently elevated levels indicate compromised renal clearance, with concentrations increasing as CKD advances (El Nahas and Bello, 2005; Fassett et al., 2011).

### 3.1 Salivary biomarker for the detection of CKD

Numerous studies have shown that saliva can serve as a non-invasive diagnostic tool for the detection of kidney disease. A variety of biomarkers associated with CKD have been identified in saliva, with the principal candidates outlined in Table 2. It is well-established that renal dysfunction affects electrolyte balance, leading to observable alterations in salivary composition. The salivary biomarkers most reported in CKD include urea, creatinine, cystatin-C, cortisol, *α*-amylase, nitrite, phosphate, and inflammatory cytokines (Celec et al., 2016). Numerous studies have indicated a notable reduction in salivary cytokine levels among individuals suffering from CKD (Thorman et al., 2010). Salivary β2-microglobulin has a very strong correlation with renal functional health condition in the diagnosis of CKD (Michelis et al., 2008). Salivary phosphate was recognized as a notably strong marker for kidney disease, observing increased phosphate concentrations in patients with the condition (Savica et al., 2007). Alterations in electrolyte profiles have also been reported, with increased salivary concentrations of calcium, sodium, and potassium observed in CKD, while bicarbonate levels remain relatively stable (Anuradha et al., 2015). Furthermore, higher levels of salivary cystatin-C were found in CKD patients when compared with healthy controls (Alsamarai et al., 2018). Consistent with these findings, other studies have reported elevated levels of immunoglobulins (IgA and IgG), albumin, and *α*-amylase in the saliva of CKD patients (Tomás et al., 2008; Pallos et al., 2015; Maciejczyk et al., 2018).

**Table 2:** Potential salivary biomarkers for detecting kidney disease.

References	Biomarkers	Category
Alsamarai et al. (2018)	Cystatin-C	Protein
Michelis et al. (2008)	β2-microglobulin	Protein
Thorman et al. (2010)	Cytokines	Proteins
Savica et al. (2007)	Phosphate	Electrolyte
Michelis et al. (2008)	Nitrite	Organic compound
Arregger et al. (2008)	Cortisol	Hormone
Anuradha et al. (2015)	Calcium, sodium, potassium	Electrolyte
Tomás et al. (2008)	Alpha-amylase	Enzyme
Maciejczyk et al. (2018)	Albumin	Protein
Pallos et al. (2015)	Immunoglobulin A and G	Antibody
Lin et al. (2022)	Saliva conductivity	Physiochemical parameter
Gull et al. (2025)	Phosphate	Electrolyte
Dental Research Journal (2026); Ahmed et al. (2015); Bader et al. (2015); Lasisi et al. (2016); Pandya et al. (2016); Patil et al. (2016); Yajamanam et al. (2016); Renda (2017)	Urea	Organic compound
Dental Research Journal (2026); Venkatapathy et al. (2014); Bader et al. (2015); Lasisi et al. (2016); Pandya et al. (2016); Yajamanam et al. (2016); Renda (2017); Temilola et al. (2019)	Creatinine	Organic compound

The literature review shows the evidence of using saliva as a diagnostic tool for identifying CKD.

Although several salivary biomarkers for CKD have been identified, the optimal markers are yet to be established. Additionally, for the detection of kidney disorders are urea and creatinine. Recent studies on the use of salivary creatinine and urea for the diagnosis of CKD were explored in the current literature (Venkatapathy et al., 2014; Ahmed et al., 2015; Bader et al., 2015; Lasisi et al., 2016; Pandya et al., 2016; Patil et al., 2016; Yajamanam et al., 2016; Temilola et al., 2019). All the studies had noted a significant increase in the levels of salivary urea and creatinine in patients suffering from CKD as compared to normal subjects. A device for the biosensing of salivary conductivity was developed by Lin et.al in 2022. A summary of few selected studies carried out for the detection of urea and creatinine levels in the saliva sample as shown in Table 3. A phosphate biomarker-based method for the early diagnosis of CKD has been proposed in the latest study carried out by Gull et al. (2025). In the study, the biosensor had a low detection limit (LOD) of 0.12 mM and worked in the linear range of 0.15-10 mM. Employing the optimized conditions, the biosensing ability of the biosensor for the non-invasive monitoring of phosphate levels in artificial saliva samples had also been explored.

**Table 3:** Summary of selected studies carried out for the detection of urea and creatinine levels in the saliva sample.

References	Sample size		Creatinine (mg/dl)		Urea (mg/dl)		Statistical method, significance
	CKD Cohort	Control group	CKD Cohort	Control group	CKD Cohort	Control group	
Lasisi et al. (2016)	50	49	2.60	0.20	92.00	20.50	Median *p* < 0.001
Venkatapathy et al. (2014)	105	37	0.660 ± 0.485	0.122 ± 0.060	-	-	Mean ± SD *p* < 0.001
Bader et al. (2015)	40	10	-	-	-	-	-
Bevc et al. (2017)	35	28	0.4489 ± 0.57186	0.1114 ± 0.13114	28.8286 ± 15.75452	21.7857 ± 11.44336	Mean ± SD *p* < 0.136 for urea *p* < 0.042 for creatinine
Pandya et al. (2016)	30	30	1.33 ± 0.94	0.08 ± 0.03	46.17 ± 11.41	12.59 ± 0.66	Mean ± SD *p* < 0.001
Ahmed et al. (2015)	160	18	2.33 ± 1.51	0.68 ± 0.18	33.05 ± 18.11	10.9 ± 4.6	Mean ± SD *p* < 0.001
Patil et al. (2016)	120	80	-	-	115.9937 ± 44.8092	41.9375 ± 15.4021	Mean ± SD *p* < 0.05 for control
Kuberappa et al. (2017)	41	41	2.20 ± 1.59	0.51 ± 0.93	110.05 ± 61.79	12.92 ± 4.78	Mean ± SD *p* < 0.00 for CKD, *p* < 0.013 (Urea). *p* < 0.049 (Creatinine)
Temilola et al. (2019)	230		11 μmol/L				Median
Yajamanam et al. (2016)	60	60	45 ± 24.9 mmol/L	9.8 ± 1.8 mmol/L	11.62 ± 0.50 mmol/L	4.48 ± 1.33 mmol/L	Mean ± SD *p* < 0.001
Chacko et al. (2024)	50		0.83 ± 0.60				Mean ± SD p – 0.52
Lin et al. (2022)	214	36	-	-	-	-	AUC ~ 0.8
Picolo et al. (2024)	10	10	11.10 ± 3.29	0.96 ± 0.18	-	-	*p* < 0.001
Ruacho et al. (2022)	84	21	-	-	-	-	-

The relationship between serum and salivary creatinine in 50 CKD patients were evaluated. Although salivary creatinine levels increased with declining kidney function, the correlation between serum and salivary creatinine was weak and not statistically significant (*p* = 0.52), indicating that salivary creatinine alone may not reliably replace serum creatinine for CKD assessment (Chacko et al., 2024).

A portable biosensor to measure salivary conductivity as a non-invasive indicator of CKD was developed. The biosensor differentiated CKD patients from healthy individuals with good diagnostic performance (AUC ≈ 0.80), suggesting that salivary conductivity could serve as a rapid screening biomarker for CKD (Lin et al., 2022). A pilot proteomics study compared saliva samples from CKD patients and healthy controls were demonstrated. CKD patients exhibited significantly elevated serum creatinine levels (11.10 ± 3.29 mg/dL vs. 0.96 ± 0.18 mg/dL, *p* < 0.001), and several differentially expressed salivary proteins were identified as potential non-invasive biomarkers for CKD diagnosis (Picolo et al., 2024).

Investigation of inflammatory biomarkers in saliva and urine of patients with systemic lupus erythematosus (SLE) and patients with lupus nephritis were studied. Salivary and urinary inflammatory markers correlated with disease activity, demonstrating the potential of saliva as a non-invasive biomarker for monitoring renal involvement. However, the study was not designed specifically for CKD diagnosis or biomarker quantification (Ruacho et al., 2022).

However, the saliva-based diagnosis has got a few drawbacks. The primary issue with salivary diagnosis is that the concentrations of certain salivary components are low in saliva compared to the blood sample. Also, levels of a few components may vary slightly within an individual from time to time. However, this variation will be normally small and will be within the normal range. Highly efficient and sensitive sensors can overcome these minor limitations.

### 3.2 Breath-based biomarker for the detection of CKD

Several studies show that exhaled breath is a rich, clinically useful, non-invasive matrix for detecting metabolic changes associated with CKD. As renal function declines, nitrogenous wastes and other metabolic by-products accumulate in blood, saliva, and are partially transferred into alveolar air; therefore, breath VOC (volatile organic compound) and targeted gas analyses can reflect CKD presence and stage. Multiple detection technologies have been applied, including sensor-based systems. The main breath biomarkers that consistently appear across studies are ammonia (NH₃) and trimethylamine (TMA). Other recurrent VOCs include acetone, isoprene, dimethyl sulfide, aldehydes and several small hydrocarbons and nitrogen-containing compounds as presented in Table 4.

**Table 4:** Potential breath biomarkers for detecting chronic kidney disease.

References	Biomarkers	Category
Bevc et al. (2017), Le Maout et al. (2018), Saidi et al. (2018), and Romani et al. (2022)	Ammonia (NH₃)	Inorganic nitrogen compound
Romani et al. (2022)	Trimethylamine (TMA)	Volatile amine (VOCs)
Saidi et al. (2018) and Romani et al. (2022)	Acetone	Ketone VOC
Romani et al. (2022)	Isoprene	Hydrocarbon VOC
Romani et al. (2022)	Dimethyl sulphide (DMS)	Organosulfur VOC
Romani et al. (2022)	Aldehydes (e.g., acetaldehyde, pentanal)	Reactive VOC / oxidative stress markers
Saidi et al. (2018)	Uremic VOCs (e.g., cyclohexanone, 2-propenal)	Uremic toxin VOCs
Marom et al. (2012), Le Maout et al. (2018), and Saidi et al. (2018)	Mixed VOC fingerprint (sensor patterns)	Multi-compound pattern

Romani et al. applied selected-ion flow-tube mass spectrometry (SIFT-MS) to the exhaled breath to quantify a panel of VOCs in real time. They found higher breath concentrations of TMA, acetone, and ammonia in CKD subjects and identified TMA, ammonia, acetone, and dimethyl sulfide as the VOCs with the best ROC performance. The study reports ammonia with a very high AUC of 0.902. It shows that breath TMA above a cutoff markedly increases CKD risk (OR ≈ 6.1), arguing that SIFT-MS is a promising clinical tool for CKD detection and staging (Romani et al., 2022). A previous study combined a commercial 6-sensor electronic nose with chemical identification to analyze the breath samples. The sensor dynamic features were extracted from sensor responses, used Principal Component Analysis (PCA), Support Vector Machines (SVMs), Hierarchical Cluster Analysis (HCA) and Partial Least Squares-regression (PLS regression), and showed the e-nose discriminated CKD, Diabetes mellitus and healthy breath patterns; gas chromatography – mass spectrometry (GC–MS) identified the VOCs behind those signatures, and a PLS model linked breath features to urinary creatinine. It is highlighted that how low-cost e-noses combined with chemometrics can noninvasively screen for CKD and associate breath signals with renal biomarkers (Lekha and Suchetha, 2021; Kalidoss et al., 2021).

A study using a variety of organically functionalized gold-nanoparticle (GNP) chemiresistors to analyze alveolar breath were conducted. By employing SVM classification alongside GC–MS for chemical analysis, they demonstrated that a selection of 2 to 3 GNP sensors could accurately differentiate early-stage CKD from healthy subjects with approximately 79% accuracy. They were also able to distinguish between stages 4 and 5 of CKD with about 85% accuracy, while a single sensor effectively separated early-stage from advanced CKD with around 76% accuracy. It was established that GNP arrays as effective and portable instruments for the staging and monitoring of CKD (Marom et al., 2012). Stable dynamic sensing capabilities and attained a diagnostic accuracy of 91% in differentiating CKD-related ammonia patterns during controlled experiments, following feature extraction and the implementation of SVM classification, and highlighted polyaniline (PANI) nanocomposites as low-cost, sensitive ammonia sensors, making them suitable for portable breath e-nose designs were exhibited (Le Maout et al., 2018). Additionally, a clinical prediction project utilized MQ-137 ammonia sensors along with non-invasive patient attributes and machine-learning classifiers to predict CKD. Although the dataset was limited, models such as Random Tree and Random Forest exhibited very high classification performance, indicating the feasibility of using low-cost breath ammonia sensing combined with machine learning. Due to a small dataset, there is a need for larger validation studies to avoid overfitting, but the results suggest the potential for simple and affordable screening devices (Garcillanosa et al., 2024).

An electrochemical gas analyzer to measure breath ammonia in a small clinical patient population was studied. The study reported significantly higher levels of breath ammonia in patients with CKD (3.32 ± 2.19 ppm) compared to controls (0.49 ± 0.08 ppm; *p* = 0.003), along with a correlated increase in sensor electric current. This study provides clinical evidence that breath ammonia correlates with advanced renal impairment and supports the use of breath NH₃ as a rapid, non-invasive marker for severe CKD (Bevc et al., 2017). In addition, the study utilized GC–MS and thermal desorption techniques to catalog various VOCs in CKD and haemodialysis patients. The researchers also reported reductions in certain VOC levels following dialysis sessions, reinforcing the connection between breath VOCs and the uremic toxin burden. Moreover, they demonstrated that GC–MS serves as the chemical ground truth for sensor selection. In a recent study, a 32-sensor electronic nose (Cyranose) was applied to analyze urine headspace samples from pregnant women across different groups, including control, high-risk, preeclampsia, and CKD (total n ≈ 95). Utilizing PERMANOVA and canonical analysis of principal coordinates, the researchers achieved group discrimination and developed a predictive CAP model that correctly classified approximately 68.4% of subjects across the groups. This study highlighted a volatilomic urinary fingerprint that may help to identify women at elevated risk for later CKD after experiencing pregnancy-related hypertensive diseases. Thus, it extends the field of volatilomics from breath analysis to urine and demonstrates the potential of electronic nose screening for CKD risk stratification (Saidi et al., 2018). A summary of select studies carried out for the detection of CKD using breath was given in Table 5.

**Table 5:** Summary of selected studies carried out for the detection of CKD using breath.

References	Sample size		Biomarker 1: Ammonia (ppm)		Biomarker 2		Statistical method, significance
	CKD Cohort	Control group	CKD Cohort	Control group	CKD Cohort	Control group	
Bevc et al. (2017)	08	06	3.32 ± 2.19	0.49 ± 0.08	Creatinine: 455.2 ± 294.1 μmol/L	Creatinine: 62.1 ± 7.5 μmol/L	Mean ± SD *p* < 0.003
Saidi et al. (2018)	16	22	Ammonia: higher in CKD AUC: ~19,000		VOC: altered CKD profile	-	-
Garcillanosa et al. (2024)	255	153	3.867 ±1.922	0.678 ±0.214	-	-	Mean ± SD p – value: 9.02096 × 10^-66^
El-Raheem et al. (2026)	42	20	VOC profile	-	2,2,6-Trimethyl-octane, 2-Butatone, 2,4-Dimethyl-heptane	-	*p* < 0.007 (2,2,6-Trimethyl-octane) p 0.028 (2-Butatone)
Romani et al. (2022)	68	54	6,450 ppbv AUC: 0.902	-	Isoprene and other VOCs: altered profile	-	*p* < 0.0001
Guo et al. (2010)	110	108	Breath ammonia	-	-	-	-
Wang et al. (2017)	55	-	Breath ammonia: positively correlated with serum creatinine	-	Product Ions: H_3_O^+^, NO^+^,and O^+^_2_	-	*p* < 0.0001
Di Gilio et al. (2024)	20	10	-	-	VOC	-	*p* < 0.05

A machine learning-based approach for predicting CKD using breath ammonia measurements combined with other non-invasive clinical attributes were evaluated. The study included 255 CKD patients and 153 healthy controls. CKD patients had significantly higher serum creatinine levels than controls (3.867 ± 1.922 mg/dL vs. 0.678 ± 0.214 mg/dL), with an extremely significant difference (*p* = 9.02 × 10^-66^). This findings demonstrate that breath ammonia, together with non-invasive clinical parameters, has the potential as a screening tool for CKD while reducing reliance on invasive blood sampling (Garcillanosa et al., 2024). A gold nanoparticle-based breath sensor array to distinguish CKD patients from healthy controls and assess disease progression using VOC profiles in exhaled breath were developed. The *p* values for various VOCs including 2,2,6-trimethyl-octane (*p* = 0.007) and 2-butanone (*p* = 0.028), highlighting the promise of breathomics as a swift, non-invasive method for screening and monitoring CKD (El-Raheem et al., 2026).

An electronic nose system for the detection of CKD through the analysis of breath ammonia were demonstrated. The sensor system effectively distinguished CKD patients from healthy individuals by analyzing exhaled breath patterns, showcasing the potential of electronic olfaction as a quick and non-invasive screening technique for kidney disease (Guo et al., 2010). Full-scan mass spectrometry to examine exhaled breath in patients with CKD were utilized. The levels of ammonia in the breath showed a positive correlation with serum creatinine (*p* < 0.0001). The use of H₃O^+^, NO^+^, and O₂^+^ product ions for ionization facilitated the estimation of clinical parameters related to CKD, underscoring the potential of breath analysis as an alternative to traditional blood-based evaluations (Wang et al., 2017). An experiment was conducted on VOCs present in exhaled breath and found VOCs as potential biomarkers for CKD. A number of VOCs showed significant differences between CKD patients and healthy individuals (*p* < 0.05), suggesting that breath metabolomics could offer a non-invasive method for the early detection of CKD and the identification of biomarkers (Gilio et al., 2024).

### 3.3 Sweat-based biomarker for the detection of CKD

Recent studies have shown that sweat, a non-invasive and easily collectable biofluid, can be an effective medium for assessing kidney function. Sweat contains various metabolites and electrolytes, and their concentration changes significantly in CKD. These constituents correlate with reduced renal clearance of uremic toxins and altered electrolyte regulation in CKD patients. Sweat is produced by eccrine glands and contains electrolytes, metabolites, hormones, and small proteins. In CKD, the kidneys have a diminished ability to filter metabolic waste, resulting in elevated levels of urea, creatinine, uric acid, potassium ions, sulphide, and lactate in sweat. This is an alternate way of toxin excretion. In research that the sweat of CKD patients holds far higher levels of potassium, urea, creatinine, lactic acid, and sulfide than in healthy individuals. Further, CKD results in disturbed energy metabolism and lower lactate dehydrogenase activity, thus making another important indicator of sweat lactate levels as well. Thus, sweat can act as a potent non-invasive diagnostic medium for real-time monitoring of kidney health. Various technological advances like electrochemical sensors, LIG circuits, and portable sweat biosensing devices developed the sensitivity for the detection of such biomarkers in human sweat, thus rendering the method of diagnosis of CKD through the help of sweat more viable. The list of commonly used sweat biomarkers for the detection of CKD is given in Table 6.

**Table 6:** List of biomarkers commonly used in sweat based CKD detection.

References	Biomarkers	Category
Kukkar et al. (2022), Huang et al. (2024), and Passornraprasit et al. (2022)	Urea, Creatinine, Uric Acid	Nitrogenous waste metabolite (Organic compound)
Kukkar et al. (2022)	Cortisol	Hormone
Kukkar et al. (2022)	Ammonia	Nitrogenous waste metabolite
Huang et al. (2024) and Kukkar et al. (2022)	Potassium (K^+^), Sodium (Na^+^), Phosphate	Electrolyte
Huang et al. (2024) and Kukkar et al. (2022)	Lactic Acid, Lactate	Metabolic biomarker (Organic acid)

Recent studies give an in-depth literature update on recent non-invasive wearable biosensors for biomarkers associated with CKD. The literature update provides information on recent developments in various biosensing approaches, such as electrochemical, optical, colorimetric, and microfluidic biosensors, that can analyze metabolites and electrolytes in bodily fluids like sweat, saliva, tear fluids, and interstitial fluid. To understand the properties of biofluids, the transport of analytes in biofluids, and the concentration variability of biomarkers in biofluids are essential. The authors further mention various critical issues associated with the development of biosensors for CKD that include biofouling, calibration issues, instability of the biosensors, and the lack of standardization between biomarkers measured in biofluids and those measured in blood serum samples. In fact, according to the literature update, most of the biosensors are still at the proof-of-concept phase and have yet to be tested on a long-term basis on the human body based on clinical studies (Kukkar et al., 2022). A *γ*-irradiation-crosslinked hydrogel comprising graphene oxide, cellulose nanofiber, and polyacrylic acid as a dual-mode platform for the detection of urea in sweat were described. Their contribution was able to integrate a colorimetric urease assay directly into the hydrogel for efficient visual analysis and further enabled LDI-MS analyses through the very same patch with improved graphene oxide properties. Their hydrogel possessed a high ability to absorb water, superior mechanical strength, and a stable colorimetric signal in urea concentrations associated with CKD. However, despite its promising sensitivity in a moderate to high concentration region, the system still suffers from limitations when detecting lower physiological levels and could be influenced by variability under real on-body sweating conditions. Their approach constitutes a truly innovative materials-driven strategy to merge Point-of-Care (PoC) readability with laboratory-grade analytical confirmation, thus standing out as a significant step forward in the direction of hybrid wearable sensors toward CKD screening (Passornraprasit et al., 2022).

Also, the prior study conducted by Huang et al. focused on the development of a wearable multi-functional biosensing platform for the analysis of sweat. Their platform relies on LIG circuitry and nanostructured electrodes printed through a laser cutter to be used for biomarkers associated with CKD determination. Their biosensing platform was comprised of LIG circuitry-based potentiometric and amperometric sensors that could detect potassium, creatinine, and lactic acid in sweat using the assistance of Cu/Cu₂O nanostructures that promote electrocatalytic properties. Moreover, their platform incorporated a hydrophilic interface created via laser cutting that could address the sweat fluidic issues associated with wearable microfluidic platforms that lead to back diffusion. However, even with its excellent analysis efficiency and scalability owing to its maskless laser fabrication technique, it still requires extensive clinical verification and long-term stability evaluation before fully applying its functionality in actual applications involving CKD patient follow-up and monitoring. At present, their portable biosensing platform can mainly detect biomarkers associated with muscle damage, exercise performance, stress recognition, and women’s health (Promphet et al., 2025). A flexible and portable biosensor of sweat for a multiplexed analysis of three major biomarkers associated with CKD: urea, creatinine, and uric acid were developed (Gao et al., 2025). The work is based on a ternary nanocomposite comprising NiCo-MOF, MWCNTs, and nitrogen-doped carbon dots. Such biosensor takes advantage of molecularly imprinted polymers for highly selective measurements and non-enzymatic stability, allowing for highly sensitive detection at remarkably low LODs and broad linear concentrations suitable for physiological sweat concentrations (Huang et al., 2024). A summary of selective studies for the detection of CKD using sweat is tabulated in Table 7.

**Table 7:** Summary of selected studies carried out for the detection of CKD using sweat.

References	Sample size		Biomarker(s)		Statistical method, significance
	CKD Cohort	Control group	CKD Cohort	Control group	
Kukkar et al. (2022)	-	-	Urea, creatinine, potassium (K^+^), sodium (Na^+^), ammonium (NH₄^+^), chloride (Cl^-^), pH, uric acid	-	-
Huang et al. (2024)	-	04	potassium ion (K+), creatinine (Cre), and lactic acid (Lac)	-	-
Vairo et al. (2017)	6	10	-	Chloride (mM) - 45 ± 7, Potassium (mM) – 5.4 ± 2.1, Sodium (mM) – 36 ± 13, pH – 7.27 ± 0.31	Mean ± SD p value – 0.56, 0.7, 0.84, 0.82
Shoumariyeh et al. (2025)	58	6	Sweat weight - adjusted R^2^ = 0.2278, *F*(2, 61) = 10.29 Sweat sodium - R^2^ = 0.3701, *F*(4, 59) = 10.26	-	*p* = 0.0001 – weight *p* = 2.261e-^06^ - sweat sodium
Gao et al. (2025)	-	-	Urea, creatinine and uric acid	-	Detectable range - 0.048 mM, 0.032 μM, and 0.024 μM
Passornraprasit et al. (2022)	-	-	Urea	-	-

A proof-of-concept study investigated the feasibility of using pilocarpine-induced sweat to estimate plasma electrolyte concentrations were performed. Sweat potassium, sodium, chloride, and pH were measured in healthy participants and compared with plasma values. No statistically significant differences were observed (*p* = 0.56, 0.70, 0.84, and 0.82, respectively), suggesting that sweat analysis may serve as a non-invasive approach for monitoring electrolyte balance, although the study was not specific to CKD (Vairo et al., 2017).

Relationship between sweat characteristics and kidney function in CKD patients were evaluated. Reduced kidney function was significantly associated with lower sweat weight (adjusted R^2^ = 0.2278, *p* = 0.0001) and altered sweat sodium concentration (R^2^ = 0.3701, *p* = 2.261 × 10^-6^). These findings suggest that sweat biomarkers may provide a promising non-invasive indicator of CKD severity and renal function decline (Shoumariyeh et al., 2025).

## 4 Review of sensory devices for non-invasive detection of CKD

Recent advancements in technology have facilitated the creation of highly sensitive and selective biosensors that can detect trace-level elements present in biomarkers linked to CKD in exhaled breath and in biological fluids such as sweat, and saliva. A biosensor serves as an analytical platform aimed at identifying or quantifying specific analytes within biological samples. The transducer converts a biochemical reaction into an electrical signal. Depending on the type of application, transducers may function through electrochemical, optical, piezoelectric, or thermal methods. The biorecognition element provides analytical specificity by selectively interacting with the target analyte. The commonly utilized receptors include enzymes, antibodies, lipid membranes, nucleic acids, and immobilized cells (Coulet and Blum, 1991). The electrical output produced by the transducer is then digitized and processed to yield a readable analytical response both biosensors and chemical sensors have been widely applied for the detection and quantification of salivary biomarkers associated with CKD (Liu et al., 2024). Among these, urea is recognized as a particularly informative biomarker for evaluating renal dysfunction (Fassett et al., 2011). Consequently, the present work focuses on monitoring urea concentration as an indicator of CKD, for which a variety of sensing devices and analytical strategies have been reported in the literature (Bertocchi et al., 1996; Eggenstein et al., 1999; Pandey, 2000; Srivastava and Kayastha, 2001; Gutiérrez et al., 2007; Yang et al., 2007).

**Figure 2:**
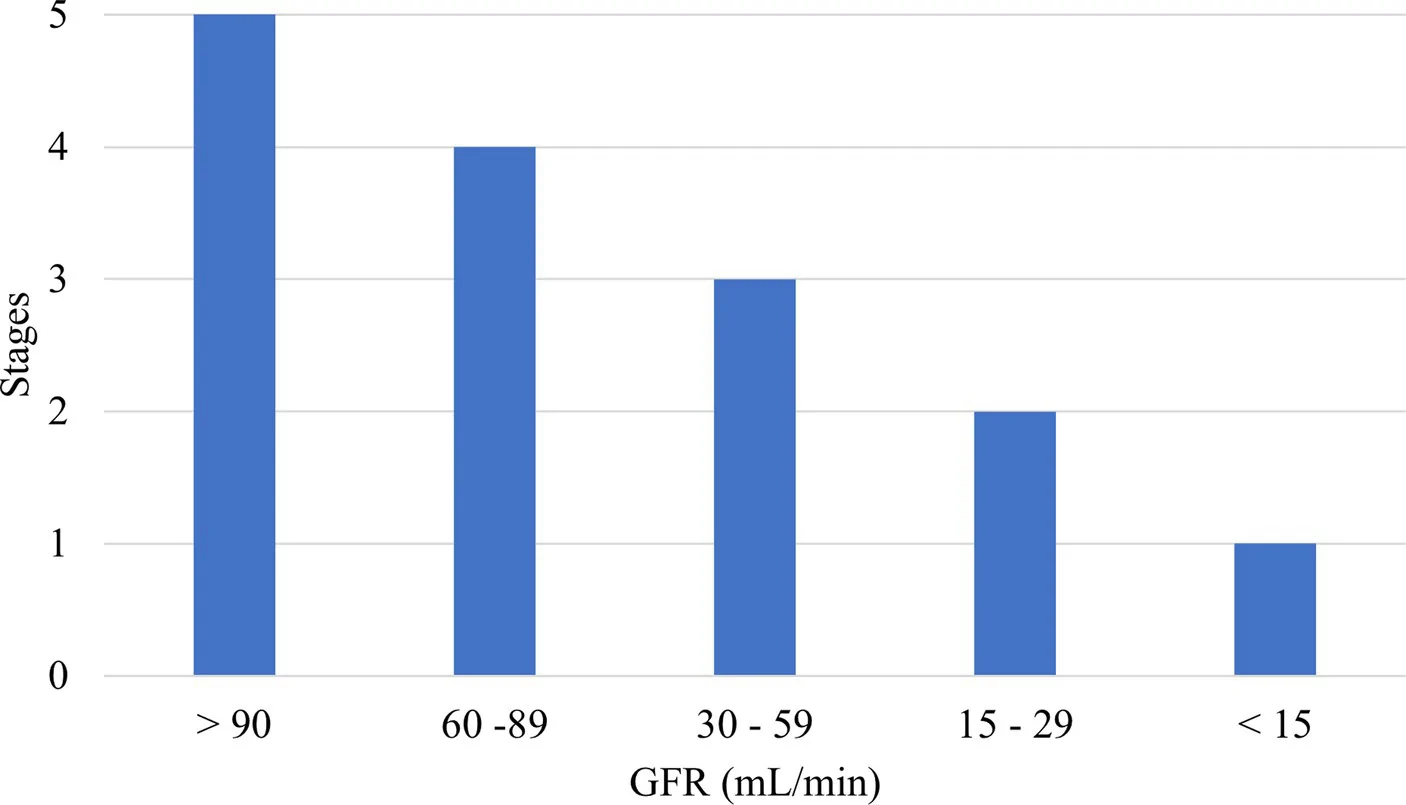
CKD stages with GFR range.

Table 8 summarizes a few works conducted on the development of urea biosensors for the assessment of diseases. Biosensors are classified based on the transducing elements and types of biorecognition elements. Biosensors based on types of transducers are electrochemical biosensors, mass-based biosensors and optical-based biosensors (Luong et al., 2008). Potentiometric, amperometric, impedimetric and conductometric sensors are the different electrochemical sensors. Potentiometric biosensors evaluate the potential difference generated between the working and reference electrode. The PoC biosensor types are categorized in Figure 2. The potential of the working electrode should depend on the concentration of the analyte in the solution.

**Table 8:** Urea biosensors and their characteristics.

References	Transducer	Technique	Application
Bertocchi et al. (1996)	Amperometric	Encapsulation technique	Saliva urea
Eggenstein et al. (1999)	Potentiometric	Entrapment polyhydrogel approach	Blood urea
Pandey (2000)	Potentiometric	Physical adsorption method	Blood urea
Srivastava and Kayastha (2001)	Potentiometric	Entrapment method	Blood urea
Yang et al. (2007)	Conductometric	Adsorption method	Urine urea
Gutiérrez et al. (2007)	Potentiometric	Covalent binding approach	Urine urea

The reference potential is provided by the reference electrode. Normally gas sensing or ion-selective electrodes are used in the potentiometric technique. Amperometric sensors function by continuously measuring current arising due the oxidation or reduction process of an electroactive species in a biochemical reaction. Conductometric biosensors can measure the changes in electrical conductivity. These sensors can measure the reactive changes occurring in a solution. Impedimetric electrochemical sensor senses the changes in the impedances. Piezoelectric biosensors are mass-based biosensor which produces an electrical signal when a mass is deposited.

The commonly used piezoelectric biosensor is the quartz crystal microbalance sensor. Optical sensors work by detecting the changes in light. These sensors are gaining importance because of their high specification, sensitivity and compact size. A wide range of sensing approaches has been reported for the detection of CKD. Desai et al. developed an ultrasensitive electrochemical sensor capable of quantifying cystatin C in urine samples, demonstrating its potential for CKD diagnosis (Desai et al., 2018). In a separate study, an automated CKD detection framework was introduced that utilized features extracted from B-mode renal ultrasound images to enable computer-assisted disease classification (Acharya et al., 2018). Ritter et al. have designed a new biosensor for quantifying the urea concentration in the body fluids (Ritter et al., 2001). Several wearable sensors are also developed for detecting CKD (Wieringa et al., 2017).

The developments in biosensor technology have advanced over the past few years. A smartphone-based optical urea biosensor was reported by Soni et al. for finding the urea levels in the saliva sample (Soni et al., 2018). In this work, a filter paper strip was used to immobilize the urease enzyme along with a pH. Urea concentration is measured by monitoring the change in the colour of the strip based on Red-Green-Blue (RGB) profiling. Optical sensors have many advantages over other sensors such as high sensitivity, specificity, and accuracy in identifying various salivary biomarkers due to their miniaturization, easy handling, and portability. At present, biosensors and fabricated sensors are increasingly employed to detect biomarkers in biological fluids. Nanomaterials are another prospect that has been incorporated into biosensors in recent times for their submicron dimensions, conductive properties and good biocompatibility (Hammond et al., 2016).

Apart from the saliva sample, other non-invasive samples for detecting CKD are urine and breath. In the urine-based analysis, normally the level of albumin in the urine is measured to analyze the functioning of kidneys. The concentration of albumin is found higher in CKD patients compared to healthy individuals (Peralta et al., 2011). The urine-based analysis is a promising approach for early identification of CKD. However, the problem with this approach is that only clinical detection methods are available for determining the albumin concentration in the urine sample.

Few research works have demonstrated the use of breath samples in CKD detection. In breath-based screening, the concentration of ammonia in the exhaled breath is monitored to predict the presence of disease (Meinardi et al., 2013). However, the level of ammonia gas in the exhaled breath is very little. This makes the method of detection hard and demands extremely sensitive sensors to measure the ammonia gas levels in the breath samples. Numerous gas analytical instruments are developed for measuring the breath ammonia concentration. Metal Oxide Semiconductor sensors are the most commonly used sensors for measuring gas concentrations (Saidi et al., 2018). These sensors work on the principle that when there is a presence of the gas molecule, it causes some changes in the electrical characteristics of the sensor as these molecules are either positively or negatively charged.

### 4.1 Biosensor standardization

A significant challenge in advancing the clinical use of non-invasive CKD biosensors is the absence of standardized procedures for sensor fabrication, calibration, and long-term stability. Differences in sensing materials, biomarker collection methods, environmental factors, and signal-processing techniques lead to varying results across different studies. Future research should focus on establishing standardized fabrication techniques, unified clinical validation processes, and multicenter evaluations to enhance reproducibility. Improvements in sensor performance may also be achieved through antifouling surface modifications, such as polyethylene glycol (PEG), zwitterionic polymers, hydrogel coatings, graphene-based nanomaterials, and microfluidic systems that minimize nonspecific protein binding and sustain sensitivity over extended use. Additionally, implementing automatic self-calibration, integrated reference electrodes, and AI-assisted algorithms for drift compensation can reduce signal drift and enhance measurement accuracy over time. Collectively, these approaches aim to support the development of effective and reliable non-invasive biosensors for the diagnosis and monitoring of CKD (Kukkar et al., 2022; Liu et al., 2024).

## 5 Machine learning algorithms with explainable AI (XAI) for early detection of CKD

Recent advancements in the technological development of artificial intelligence paves the way for early detection and initiating timely interventions. Machine learning (ML) and Deep Learning (DL) techniques are emerging as powerful tools for automated disease prediction and diagnosis. In this work, we have analysed various ML algorithms in the detection of CKD and non-CKD. The models investigated included are K-Nearest Neighbours (KNN), Decision Tree (DT), RF, SVM, and Convolutional Neural Network (CNN).

DT method is a technique used for classification and regression. It is a way to predict both discrete and continuous characteristics (Ahmed et al., 2022; Lasisi et al., 2016). KNN supervised classifier algorithm that works by identifying ‘K’ nearest data points based on Euclidean distance. SVM is a binary classifier that will classify the data set by marking the datasets as positive and negative sets. The support vectors are defined based on the given data sets, and the feasibility of the hyper-plane is derived. The SVM classifier determines the best hyper-plane such that the boundary of the division line is maximized to separate the defined support vectors. Next, the remaining samples are tested by the SVM model for predicting their class. SVMs are intrinsically binary classifiers. Since classification is performed on more than two classes, a multiclass classification strategy is adopted. The most popular ones are one-against all (OAA) and one-against-one (OAO). Both strategies lead to similar results in terms of classification accuracy. OAA strategy is used for classification since it involves a reduced number of binary decompositions. The conventional CNN model is similar to the Artificial Neural Network (ANN), and it is primarily used for the processing of images. In order to process the sensor response, a one-dimensional CNN is used. The benefit of using CNN compared to its predecessors is that it will automatically extract the best features without the help of a separate feature extraction algorithm. Therefore, we do not have to apply two different algorithms for feature extraction and classification. This helps to reduce the computational complexity and simulation time of the network. CNN is computationally more effective compared to traditional learning approaches.

Some works in CKD predictions using the machine learning technique have established sophisticated models with high diagnostic proficiency. It compares classifiers like RF, SVM, KNN, DT, and Naive Bayes (NB), with RF achieving up to 100% accuracy and KNN reaching 98-99% on UCI CKD datasets with 25 attributes. The authors highlight the potential of machine learning to decrease the time required for diagnosis, enhance the accuracy of early detection, and facilitate personalized treatment planning for patients with chronic kidney disease (Nimmagadda et al., 2023). A pipeline web application for CKD prediction, referred to as Machine Learning-based Chronic Kidney Diseases Prediction (ML-CKDP), has been developed. This approach incorporates thorough preprocessing and feature selection, which includes the transformation of nominal input variables, imputation of missing data, and scaling using Min-Max scaling (Halder et al., 2024). Feature selection methods such as correlation, chi-square, and Lasso are also assessed, with performance analysis using classifiers like RF and AdaBoost, which attained a perfect score. Though its application orientation and deployment emphasis can practically be considered an advantage, some disadvantages also exist, including using small and imbalanced datasets. Nevertheless, the authors highlighted these disadvantages, suggesting the web application is a precursor that can be utilized by physicians (Gao et al., 2025). The authors propose the use of Chi^2^-MI, a hybrid feature selection method that integrates the Chi^2^ Test with Mutual Info, eliminating redundant characteristics from the UCI CKD dataset. The authors then extensively preprocess the data by applying encoding, KNN Imputation, removing outliers by the use of the Louder approach, and applying SMOTE. The contribution of the study is the reproducible feature selection, with the extensive comparison of the proposed algorithms. The limitations or drawbacks include the reliance on the small UCI dataset (Dey et al., 2022).

A study on Explainable Machine Learning for CKD by Ghosh and Khandoker (2024) involved model training using techniques like Logistic Regression (LR), RF, DT, NB, and XGBoost on a dataset of 491 patients. They were focusing on interpretability through SHapley Additive exPlanations (SHAP) and Local Interpretable Model-agnostic Explanations (LIME). They concluded that the model that performed the best was XGBoost, with an AUC value of about 0.969 and an accuracy of 93%. SHAP values helped reveal the most important factors, like creatinine, HbA1c, and Age for the model’s outputs, while LIME explained model outputs at the individual level. The originality in the study consists of the solution integrating model interpretability techniques with its good performance capabilities. The study faces issues related to the generality of the dataset, which consists of only 491 patients. They suggest future studies for its validation across multiple centres and the incorporation of Explainable AI into practices. They examined whether these feature sets can be applied using machine learning algorithms to classify CKD and potentially predict creatinine levels. The authors compared how well RF and ANN models can be applied to a 400-sample database segmented into three groups based on feature accessibility levels. Their main contribution is useful, focusing on how RF is better suited to home analysis (± 92.5% accuracy) and how both RF and ANN classify above 98% using lab data, while creatinine regression is significantly aided by lab data (Metherall et al., 2025).

The classifiers in machine learning for CKD and design of a hybrid machine learning model that incorporates the Pearson correlation in the process of feature selection were analyzed. The authors employ the Gaussian naive bayes, Gradient Boosting (GB), and DT algorithms and apply a RF algorithm as the meta-classifier to deliver 100% accuracy on the UCI dataset. This paper underlines the significance of the selection of the learners to avoid overfitting and the challenges involved in the small public datasets. Challenges faced by small public datasets are mentioned in this paper, and the paper underlines the significance of proper validations to avoid overoptimistic outcomes (Khalid et al., 2023). The use of machine learning techniques for the prediction of CKD, finding that the best-performing one is XGBoost with an accuracy rating of 98.3%, although this is enhanced by the use of PCA were compared. They retain 25 variables that are then reduced by 30% through PCA and feature selection for the evaluation of 12 machine learning techniques. The use of PCA in machine learning applications is brought into focus. The drawbacks include the use of UCI-style datasets and the lack of external validation. It nevertheless well represents the effectiveness of XGBoost for tabular CKD problems (Islam et al., 2023).

A survey and experiment on the UCI CKD dataset with the implementation of the following machine learning algorithms on the data: ANN, C5.0, CHAID, LR, LSVM (L1 and L2), and Random tree were performed. They analyze the approaches for the feature selection process, such as correlation, wrapper, and LASSO, as well as oversampling approaches such as SMOTE. They also demonstrate the efficiency of their Deep Neural Network (DNN) approach with an accuracy level of 99.6 percent. They point out the influence of the preprocessing step on the accuracy of the algorithms supported by a comparative analysis (Chittora et al., 2021). Comparison of several classical machine learning methods, including KNN, DT, RF, and ensemble methods, thereby achieving a precision rate of about 99.16% with ensemble/tree-based approaches were introduced. They had emphasised on multi-class staging and translating model outputs into actionable clinical guidance. The results further underline that the ensemble and tree methods perform very well with tabular medical data. It also shows that multi-class metrics such as precision and recall are more important instead of just binary accuracy. Similar to other works, they note that the next steps involve external validation and prospective testing (Sharma et al., 2025).

Feature selection and classification techniques with SMOTE for class imbalance and SHAP for interpretation were compared. They report nearly 100% for specific sets and around 92% for multi-class staging for XGBoost and genetic algorithms. Their work is related to model interpretation and interpretability regarding features, though the high levels of accuracy must be considered in relation to the limited dataset employed (Gogoi and Valan, 2025). A deployment solution by developing a robotic process automation mobile application that combines several models for machine learning models, such as LR and RF were described. They describe two ensemble techniques, MKR Stacking and MKR Voting, indicating that MKR Stacking results in 99.5% accurate results. Their work requires overcoming implementation complexities as well as regulations for real-world implementation for validation over a long term (Bijoy et al., 2025). Supplementary Table 1 shows the studies carried out for the detection of CKD using machine learning approaches. CMQMC-RBEL framework, which integrates quantum mutual information-based feature selection with Radial Basis Extreme Learning for CKD prediction using the UCI CKD dataset were proposed. The model achieved 98% accuracy, demonstrating the effectiveness of quantum-inspired feature selection for binary CKD classification, although validation was limited to a benchmark dataset (Jayashree and Anitha, 2025). Combination of transcriptomic datasets, network toxicology, and machine learning to identify molecular mechanisms and pathogenic targets associated with plasticizer-induced CKD were performed. RF and stepwise LR identified key biomarkers, that require further experimental and prospective clinical validation (Mao et al., 2026).

Enhanced GRU (EGRU) model, optimized using Improved Clouded Leopard Optimization and stacked autoencoder-based feature extraction, achieved 98.75% accuracy on the UCI CKD dataset were proposed. The proposed model has excellent classification performance but lacks external validation on real-world clinical datasets (Srirenga Nachiyar and Marichamy, 2026). Comparison between the MLFIS and ANFIS models for the early diagnosis of CKD utilizing anonymized clinical records were performed. Both models demonstrated exceptional performance, achieving accuracy rates exceeding 99%. This indicates that fuzzy inference systems can significantly aid in clinical decision-making. However, further validation across a wider range of hospitals is necessary (Sharma et al., 2026). A deep learning framework incorporating Grad-CAM interpretability for the diagnosis of CKD using CT images were demonstrated. The model exhibited encouraging diagnostic capabilities and improved explainability. The evaluation was restricted to benchmark data and did not include external clinical validation (Panse et al., 2025). Creating AI models to assess the five-year mortality risk among individuals suffering from CKD were studied. The top-performing model recorded an F1-score of 96.35% and an AUROC of 0.941, showcasing its robust prognostic potential, yet it also pointed out the importance of conducting prospective external validation (Nopour and Shanbehzadeh, 2026).

Various machine learning algorithms for the diagnosis of CKD utilizing the UCI dataset were analyzed. The RF algorithm attained the highest performance with an accuracy of 99%, highlighting the efficacy of ensemble learning in automated CKD screening, even in the absence of multicentre validation (Mehla et al., 2026). Multiple machine learning algorithms with SHAP/LIME explainability and several feature selection techniques across three CKD datasets were integrated. The framework achieved up to 100% accuracy with SVM while providing interpretable predictions to support clinical decision-making (Huda et al., 2026). A large retrospective study for a machine learning model for CKD diagnosis in high-altitude populations using demographic and laboratory biomarkers were developed. The model achieved an AUC of 0.87, demonstrating good diagnostic performance, although validation was limited to a single geographic region (Liu et al., 2026). Transcriptomic analysis, machine learning, and experimental validation to identify N6-methyladenosine (m6A) RNA methylation-related biomarkers for CKD were proposed. Diagnostic signatures achieved AUC values between 0.91 and 0.97, supporting their potential utility for risk prediction and immune subtype classification (Chen et al., 2026). XGBoost-based model to predict CKD progression in older adults and externally validated it across multiple ethnic cohorts were developed. The model achieved ROC-AUC values of approximately 0.89–0.93, demonstrating strong generalizability for progression risk prediction (Wang et al., 2026).

A hybrid feature selection strategy with an ensemble deep learning architecture achieved 99.75% accuracy for binary CKD classification on the UCI dataset were proposed. The results highlight the benefit of combining filter and wrapper feature selection methods, although external clinical validation is still required (Yogesh et al., 2025). A DNN to predict mortality among CKD patients using electronic health records were developed. The deep learning model outperformed RF and XGBoost in discrimination (AUC = 0.83), demonstrating its potential for personalized mortality risk assessment despite the retrospective study design (Kim and Woo, 2025).

## 6 Discussion

**Figure 3:**
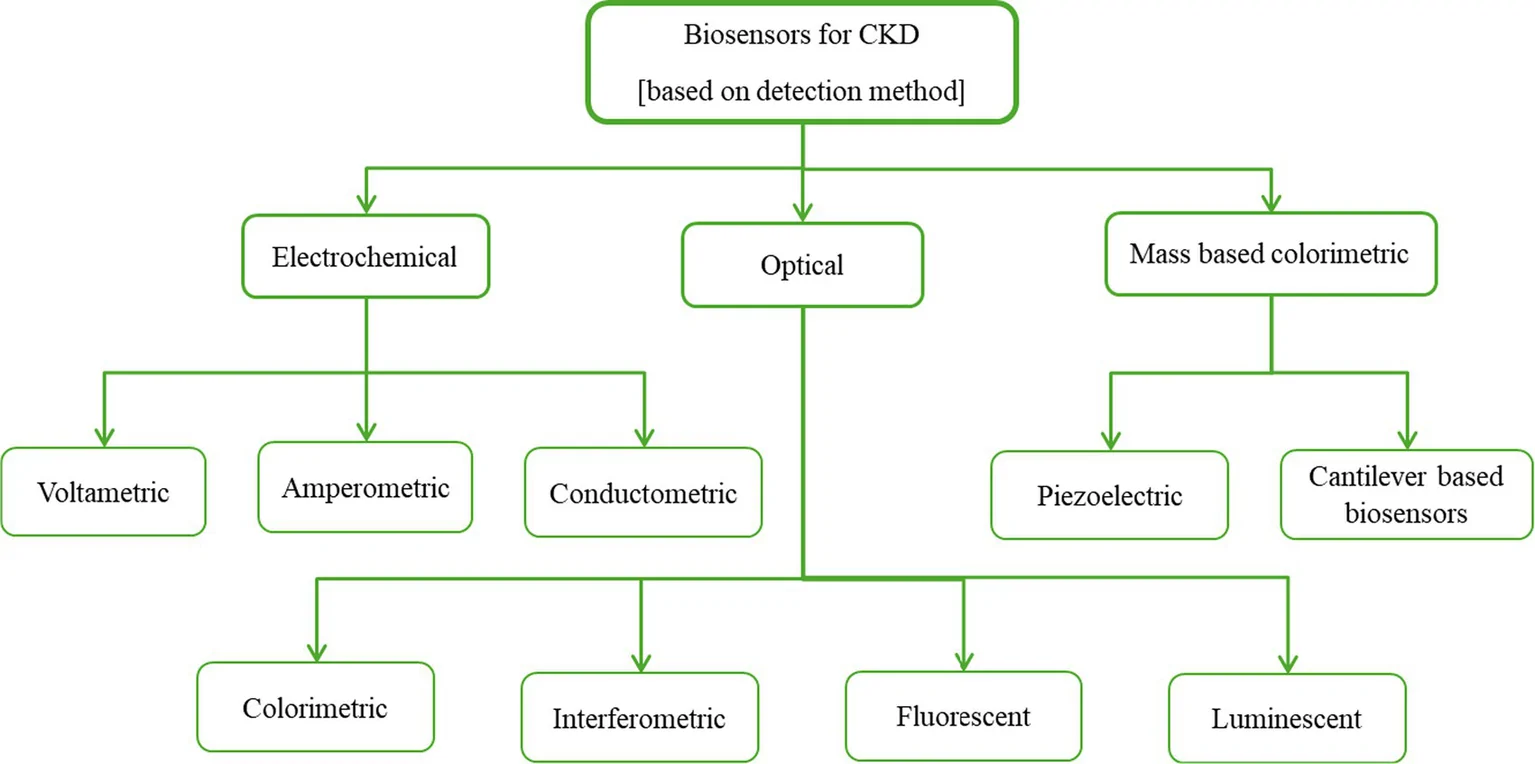
Types of PoC biosensor.

The development of various non-invasive sensory devices can help in early diagnosis of chronic kidney disease and can be used in wearables to monitor continuously. There are two components in such integrated systems namely the sensor device and the integrating machine learning model. The integration of machine learning models to sensor response will automate the diagnosis of CKD. Figure 3 shows the AI integrated biosensing workflow.

**Figure 4:**
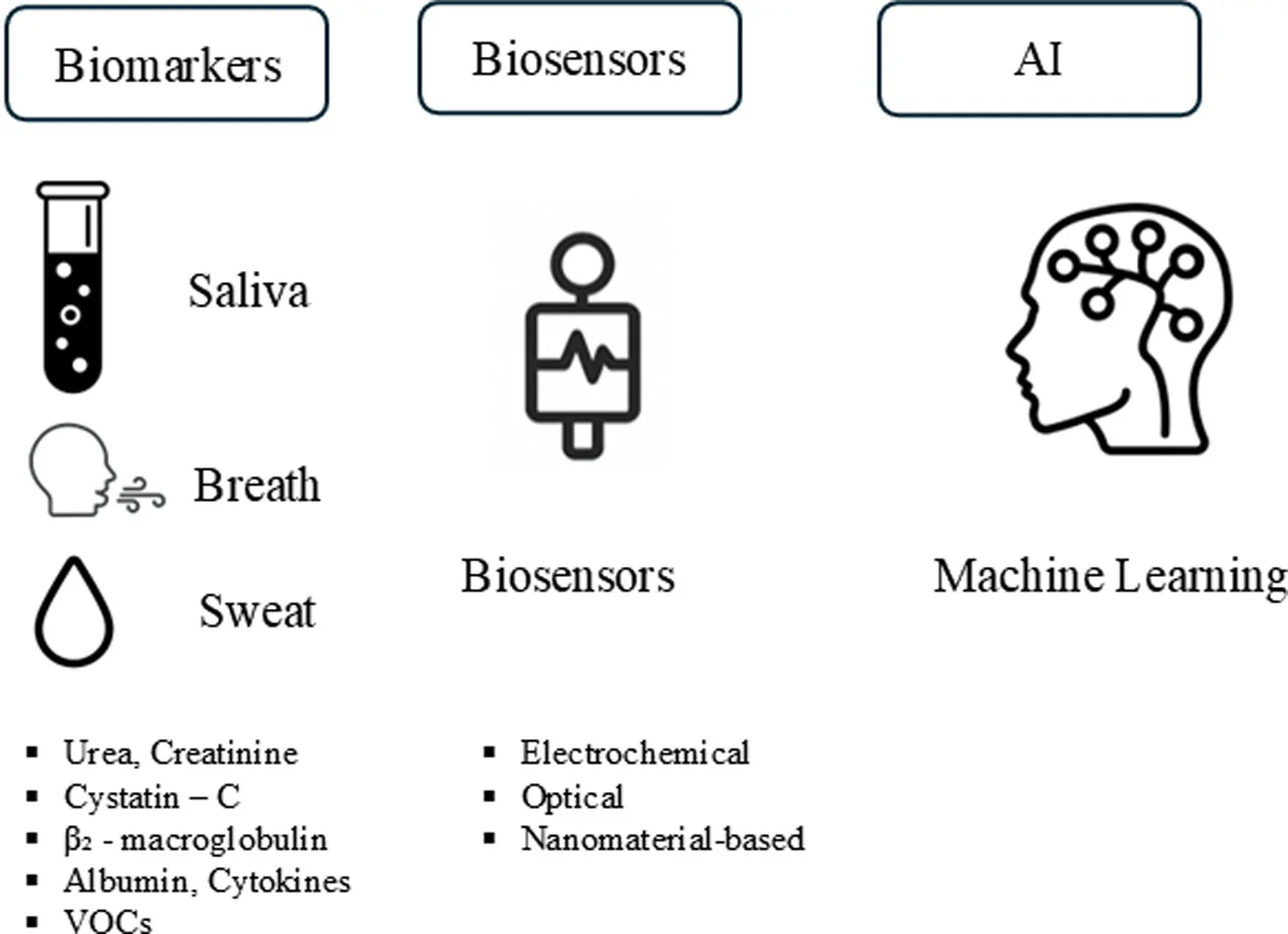
AI-integrated biosensing workflow.

Saliva based biosensor was used for detecting urea level, and different algorithms were applied to evaluate the performance of the implemented models. The majorly used biomarkers for the diagnosis of CKD in the references are given in Figure 4. The major performance parameters are computed. All the parameter values are calculated from the elements of the computed confusion matrix. Figure 4 compares the performance of all the implemented techniques. Singular Value Decomposition and PCA algorithms are feature extraction techniques, whereas KNN is a supervised classifier like SVM. SVD-SVM, PCA-SVM and PCA-KNN are examples of traditional machine learning techniques. In these three models, two separate algorithms are used for feature extraction and classification. The SVD-SVM, PCA-SVM and PCA-KNN networks have predicted the sample with an accuracy of 88.79, 87.32 and 84.51%, respectively. The RNN model is usually used for sequence prediction problems. CNN is a deep learning network that automatically extracts the features from the input signal. Therefore, CNN-based networks have the potential to give better efficiency compared to traditional machine learning models. The CNN model with a single hidden layer is referred to as shallow CNN. The single-layer CNN attained a classification accuracy of 91.55%. CNN combined with RF network classified the test samples with an accuracy of 90.59%.

The comparative analysis of non-invasive biofluids for the detection of CKD emphasizes the diagnostic importance of biomarkers found in saliva, sweat, and breath, as well as hybrid multimodal approaches. Saliva-based biomarkers, including urea, creatinine, uric acid, sodium, potassium, and pH, have demonstrated strong predictive capabilities. Machine learning models such as SVM, RF, and ANN have achieved receiver operating characteristic area under the curve (ROC–AUC) scores ranging from 0.85 to 0.94. Further performance improvement is observed when saliva is analyzed with a multi-biomarker panel with advanced models such as RF and XGBoost, with some papers achieving an AUC as high as 0.96. Biomarkers associated with sweat, such as urea, creatinine, and several electrolytes, possess slightly lower yet equally robust diagnostic performance, reporting ROC-AUC performance between 0.80 and 0.90 with SVM and ANN. In this context, integration with wearable sweat sensors significantly improves the predictive power of developed models, with an RF and CNN output that achieves AUC values in the range of 0.88-0.95.

Although several machine learning models have reported exceptionally high diagnostic accuracies (99–100%) for CKD prediction, particularly when evaluated using relatively small datasets such as the UCI CKD dataset, these findings should be interpreted with caution. Since the UCI CKD dataset is a binary class confined to a small demographic. There is a possibility of overfitting and data leakage. Limited diversity among patients in small datasets increases the chance of overfitting because models may pick up on patterns that are only relevant to that dataset rather than associations that are applicable in a clinical context. This will lead to limiting their ability to apply to new, independent patient groups. Another critical concern is the occurrence of data leakage, which refers to the situation where information from the testing dataset unintentionally influences the training of the model or the selection of features, resulting in excessively optimistic evaluations of performance. Frequent triggers of data leakage involve executing feature selection, normalization, or imputation of missing values before the dataset is split into training and testing groups. Such procedures could artificially inflate performance indicators, thus compromising the reliability of the findings reported. Hence a dataset with more data instances, more features, broad category of people are required before coming to a conclusive CKD prediction.

In upcoming studies, it is essential to adopt rigorous machine learning processes by segregating data preprocessing within each training iteration, employing nested cross-validation when appropriate, and testing models on separate external datasets acquired from multiple healthcare facilities. Furthermore, thorough documentation of preprocessing strategies, hyperparameter optimization, and reproducible validation processes will be imperative for developing clinically trustworthy AI models aimed at diagnosing CKD.

**Figure 5:**
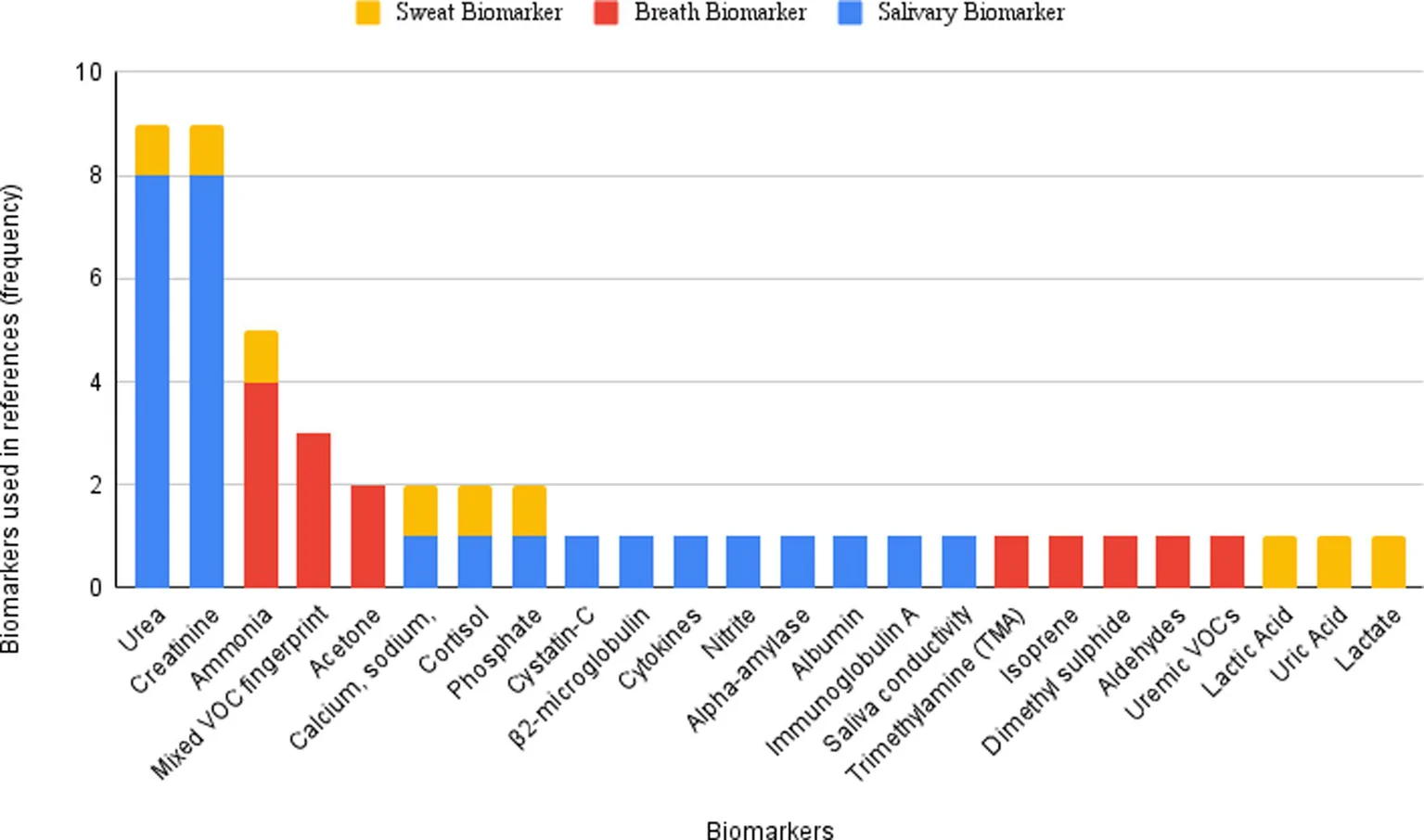
List of biomarkers (sweat, breath, salivary) used in literature study.

**Figure 6:**
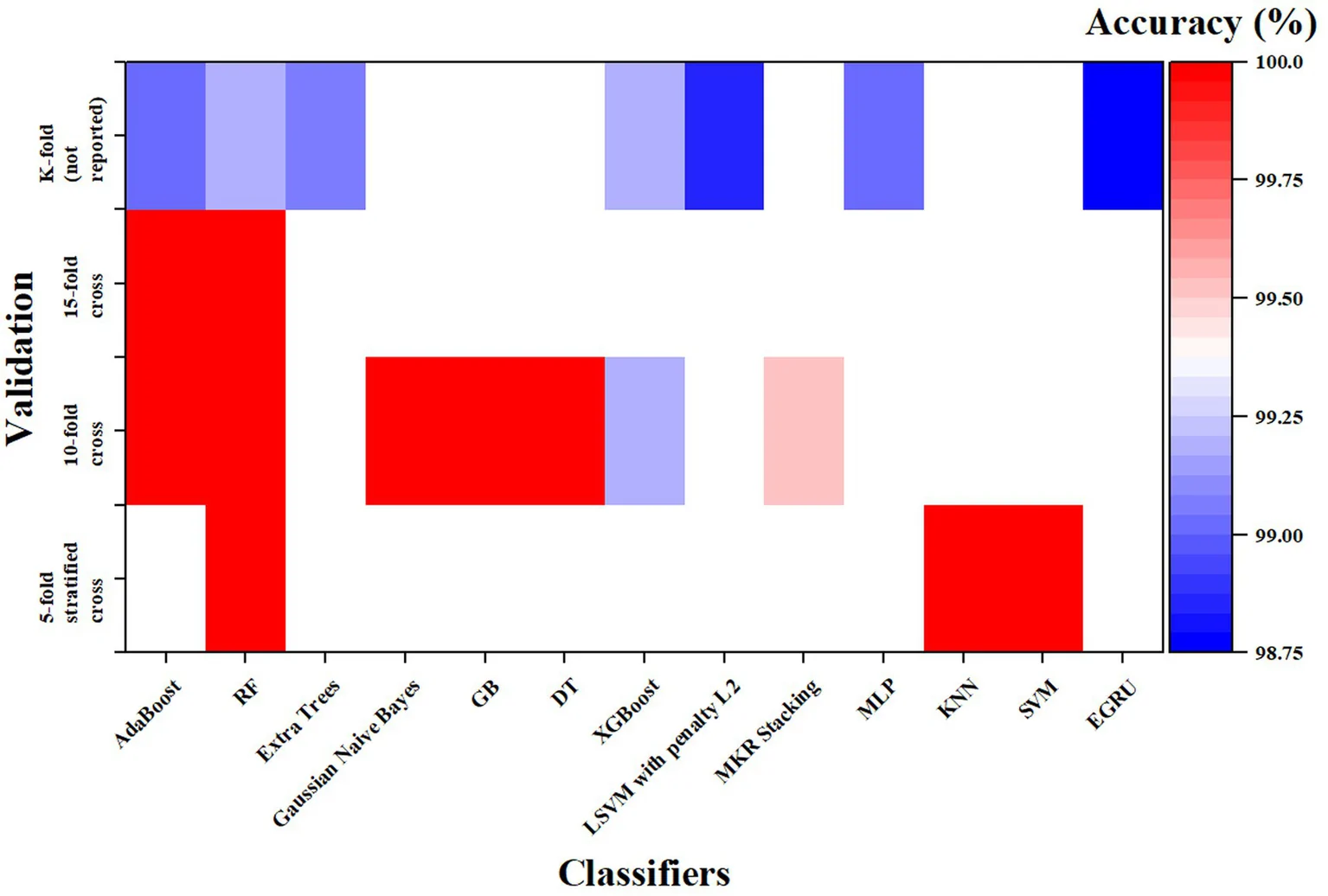
Heat map analysis of various classifiers across validation and their accuracies for UCI repository.

Table 9 presents a summary of machine learning models developed using the UCI Chronic Kidney Disease dataset. The majority of studies utilized 10-fold cross-validation, with some studies used 5-fold stratified cross-validation, 15-fold cross-validation, and k-fold cross-validation (with the number of folds not specified). The classifiers assessed include RF, AdaBoost, XGBoost, Gaussian Naïve Bayes, GB, DT, KNN, SVM, MLP, LSVM with L2 penalty, MKR Stacking, Extra Trees, and EGRU. The reported accuracies varied from 92.5 to 100%, with several studies reporting 100% accuracy for models such as RF, AdaBoost, KNN, SVM, Gaussian Naïve Bayes, GB, and DT. However, these high accuracy rates should be interpreted with caution, as many of these studies were based on the relatively small UCI dataset, which might increase the risk of overfitting and restrict the applicability to external clinical datasets. Figure 5 displays a heat map that illustrates the relationship between validation strategies, classifiers, and their reported accuracies for machine learning models created with the UCI CKD dataset. Warmer colors (red) indicate higher classification accuracies, while cooler colors (blue) signify lower accuracies. The heat map reveals that 10-fold cross-validation was the most commonly used validation strategy, showing consistently high performance across various classifiers. RF and AdaBoost exhibited high accuracies with different validation methods, while KNN and SVM reached 100% accuracy under 5-fold stratified cross-validation. Models assessed using k-fold cross-validation (number of folds not specified) generally achieved accuracies ranging from 98.75 to 99.17%. Overall, the figure emphasizes the prevalent use of cross-validation and the consistently high performance reported by several classifiers on the UCI CKD dataset. Figure 6 illustrates a Heatmap analysis comparing the classification accuracy (%), across various classifiers and validation techniques for UCI-CKD dataset.

**Table 9:** Summary of selected studies validation, classifiers accuracy related to the machine learning prediction model for UCI repository.

Dataset	Model	Validation	Classifier	Accuracy (%)
UCI repository	Machine learning prediction	10-fold cross	AdaBoost	100
UCI repository	Machine learning prediction	15-fold cross	RF	100
UCI repository	Machine learning prediction	10-fold cross	RF	100
UCI repository	Machine learning prediction	15-fold cross	AdaBoost	100
UCI repository	Machine learning prediction	k-fold (not reported)	Extra Trees	98
UCI repository	Machine learning prediction	10-fold cross	RF	92.5
UCI repository	A hybrid model, type of ensemble technique in stacking	10-fold cross	Gaussian Naive Bayes	100
UCI repository	A hybrid model, type of ensemble technique in stacking	10-fold cross	GB	100
UCI repository	A hybrid model, type of ensemble technique in stacking	10-fold cross	DT	100
UCI repository	A hybrid model, type of ensemble technique in stacking	10-fold cross	RF	100
UCI repository	Machine learning prediction	10-fold cross	XgBoost	99.2
UCI repository	Machine learning prediction	k-fold (not reported)	LSVM with penalty L2	98.86
UCI repository	Machine learning prediction	k-fold (not reported)	XgBoost	99.06
UCI repository	Machine learning prediction	k-fold (not reported)	Extra trees	99.06
UCI repository	Machine learning prediction	k-fold (not reported)	RF	99.17
UCI repository	Machine learning prediction	k-fold (not reported)	XgBoost	99.17
UCI repository	Machine learning prediction	10-fold cross	MKR Stacking	99.5
UCI repository	Machine learning prediction	k-fold (not reported)	AdaBoost	99
UCI repository	Machine learning prediction	k-fold (not reported)	MLP	99
UCI repository	Machine learning prediction	5-fold stratified cross	RF	100
UCI repository	Machine learning prediction	5-fold stratified cross	KNN	100
UCI repository	Machine learning prediction	5-fold stratified cross	SVM	100
UCI repository	Machine learning prediction	k-fold (not reported)	EGRU	98.75

Breath-based assessment also provides considerable promise. Ammonia concentration, which is analyzed through SVM and LR, provides ROC-AUC values between 0.86 and 0.93, while pattern analysis of VOCs using RF, ANN, and deep learning results in higher diagnostic performances, in the range of 0.90-0.97. The highest diagnostically developed performance is reported within hybrid non-invasive approaches that combined biomarkers from both saliva and breath, where models such as XGBoost and RF produce near-clinical ROC-AUC values between 0.93 and 0.98 (Islam et al., 2023; Ghosh and Khandoker, 2024; Gogoi and Valan, 2025; Li et al., 2025). These observations are also reflected in the ROC-AUC comparative graph, where it can easily be inferred that ensemble techniques such as RF + XGBoost, standalone XGBoost, and deep learning models perform better compared to all other techniques, reaching AUC scores very close to 0.98-0.99. RF and SVM classify well, whereas basic models such as LR perform moderately well. In the comparative graph, it can easily be inferred that state-of-the-art machine learning techniques, especially those related to boosting and deep learning, possess better discriminatory ability for identifying CKD using a wide range of non-invasive biofluids. It can thus be inferred from this table and graph that non-invasive biofluids, along with state-of-the-art machine learning models, possess great diagnostic efficacy and are suitable alternatives to blood-based diagnosis of CKD.

Some of the limitations of serum-based diagnostic approaches will limit their wide use in clinical settings. The primary issue with salivary diagnosis is that the concentrations of certain salivary components are low in saliva compared to the blood sample. Also, levels of a few components may vary slightly within an individual from time to time. However, this variation will normally be small and will be within the normal range. Highly efficient and sensitive sensors can overcome these minor limitations. There is a need for ultra-sensitive detection systems for low-concentration gases such as NH₃ in breath. Stimulation of sweat is also a limitation in sweat-based sensors. Above all, there are challenges in large-scale clinical validation for non-invasive approaches.

Although the reported studies demonstrate encouraging results for AI-assisted non-invasive diagnosis of CKD, several methodological limitations should be acknowledged. Many studies were conducted using relatively small sample sizes, particularly those involving saliva, breath, and sweat biomarkers, where patient cohorts often comprised fewer than 100 participants. The use of limited sample sizes diminishes statistical power and may heighten the risk of overfitting, particularly in the context of developing machine learning models. Moreover, a significant number of AI-focused studies have depended on publicly accessible datasets, such as the UCI Chronic Kidney Disease dataset, which may not sufficiently capture the diversity of real-world CKD populations regarding ethnicity, age, disease stage, comorbidities, and healthcare environments. The prevalence of single-centre datasets and the lack of extensive external validation further limit the generalizability of the models reported. While numerous studies have indicated high diagnostic accuracies (often surpassing 95%), these findings should be approached with caution due to the frequent absence of independent multicentre validation. Future investigations should emphasize large-scale, prospective, multicentre studies that adhere to standardized protocols for biospecimen collection and biomarker analysis. Furthermore, external validation involving geographically and demographically varied populations is crucial to confirm the robustness, reproducibility, and clinical relevance of AI-driven non-invasive CKD diagnostic systems.

A significant obstacle in the creation of effective AI models for non-invasive CKD diagnosis is the scarcity of extensive, diverse, and well-annotated clinical datasets. Numerous existing studies depend on small, unbalanced, or single-center datasets, which heightens the risk of overfitting and restricts the generalizability of predictive models. Consequently, future research should investigate Federated Learning (FL), which allows for collaborative model training across various healthcare institutions without necessitating the exchange of sensitive patient information, thus safeguarding privacy while enhancing model robustness and diversity. Furthermore, techniques for Synthetic Data Generation, utilizing Generative Adversarial Networks (GANs) and other generative AI methodologies, can be applied to augment minority classes, tackle class imbalance, and enhance model training in situations where patient data is limited. However, it is crucial that synthetic datasets undergo thorough validation to confirm that they accurately maintain clinically relevant biomarker distributions and do not introduce artificial bias. The combination of FL with synthetic data generation holds the promise of advancing the development of more dependable, privacy-preserving, and clinically generalizable AI models for non-invasive CKD diagnosis. The development of Agentic AI offers a potential advancement in the field of non-invasive CKD diagnosis. Unlike traditional AI models, Agentic AI systems are capable of independently interpreting clinical data, executing multi-step reasoning, retrieving pertinent medical information, and aiding in decision-making that adapts to clinician input. These features could enhance the effective integration of various biosensor data, ongoing patient monitoring, customized risk evaluation, and responsive clinical decision support. When utilized alongside Federated Learning and synthetic data generation, Agentic AI may contribute to the creation of more dependable, privacy-conscious, adaptable, and clinically applicable AI models for non-invasive CKD diagnosis.

## 7 Future perspectives

The progress of PoC devices, along with the incorporation of biosensors into wireless communication and data analytics systems, is anticipated to facilitate smooth data transmission and remote health monitoring. This will allow for prompt clinical intervention, ongoing patient surveillance, and tailored therapeutic approaches. The integration of artificial intelligence will improve the interpretation of sensor data and enhance risk prediction. Even though many non-invasive sensors using different detection methods have been developed, each has its own limits in a clinical setting. The following approaches can address the issues and pave the way for future research directions.

### 7.1 Impacts on external factors affecting the sensors performance

The use of single sensor may have less sensitive response if there is low concentration of the serum used for diagnostic applications, incorporating an array of sensors will help to overcome such measures. The effect of humidity and temperature variation will also cause a variation in sensor performance. Incorporating humidity and temperature could enable to track the variations from time to time.

### 7.2 Patient-centric approaches and personalized care

The adoption of patient-centric strategies involves the development of customized predictive and interactive models that enhance collaboration between patients and healthcare professionals. Nevertheless, the effective implementation of these strategies into standard clinical practice continues to pose difficulties, mainly because of constraints in ongoing patient-clinician communication and the lack of cohesive management systems. Recent developments in the Internet of Things (IoT) and the Medical Internet of Things (MIoT) present encouraging solutions by facilitating continuous, worldwide connectivity within healthcare networks. These innovations enhance the provision of personalized care through better monitoring, prompt interventions, and more efficient long-term management of diseases.

### 7.3 Advanced data processing and predictive algorithms

The suggested framework that integrates sensors generally consists of two main components: a sensing unit that handles data acquisition and a data processing and classification module that facilitates analytical interpretation. Widely used machine learning methods for feature extraction and classification encompass PCA, DT, Linear Discriminant Analysis, and SVMs. Although these traditional algorithms provide various benefits, their application in real-time sensing scenarios poses significant challenges. These challenges include the selection of suitable feature extraction techniques, the reduction of computational complexity and processing time, and the assurance of robust and reliable detection performance. To overcome these challenges, the automated interpretation of sensor outputs can be improved through the use of advanced deep learning models, provided that issues related to overfitting and underfitting are properly addressed.

### 7.4 Policies and regulatory requirements for AI-integrated biosensing devices

Although AI-integrated biosensing systems show great potential for diagnosing chronic kidney disease, their implementation in standard clinical practice necessitates adherence to rigorous regulatory standards. Regulatory bodies, including the U. S. Food and Drug Administration (FDA) and the European Union under the Medical Device Regulation (EU MDR 2017/745), along with CE marking, and the Central Drugs Standard Control Organization (CDSCO) in India, require proof of analytical validity, clinical validity, and clinical utility prior to the approval of AI-enabled diagnostic devices for clinical application. Beyond proving high diagnostic accuracy, sensitivity, and specificity, manufacturers are obligated to demonstrate the safety, reliability, reproducibility, and robustness of both the sensing platform and the AI algorithms through multicenter clinical validation studies. Additionally, AI models must be assessed for transparency, interpretability, cybersecurity, bias mitigation, data privacy, and performance across varied patient demographics. Ongoing monitoring and regular updates of adaptive machine learning algorithms must align with regulatory guidelines to maintain consistent performance throughout the device’s lifecycle. Moreover, compliance with quality management standards such as ISO 13485 for medical devices and ISO 14971 for risk management is crucial during the development of the device. By addressing these regulatory, ethical, and quality assurance criteria, the successful transition of AI-integrated non-invasive biosensors from research settings to everyday clinical practice can be achieved, thereby enhancing clinician and patient trust in these technologies.

Explainable Artificial Intelligence (XAI) is set to be pivotal in promoting the clinical integration of AI-enhanced non-invasive CKD sensing systems. Although sophisticated machine learning and deep learning models frequently demonstrate exceptional predictive accuracy, their opaque ‘black-box’ characteristics can undermine clinician trust and obstruct regulatory approval. Consequently, future studies should integrate interpretable AI methodologies such as SHAP and LIME to assess the impact of individual biomarkers and sensor attributes on each diagnostic prediction. Additionally, feature attribution techniques can pinpoint the most significant biomarkers, allowing clinicians to confirm that the AI model’s needs align with established clinical practices. Beyond interpretability, it is essential to evaluate model calibration to ensure that predicted probabilities genuinely represent the actual likelihood of disease, thus enhancing the reliability of clinical decision-making. Moreover, uncertainty estimation methods, such as Bayesian neural networks, Monte Carlo dropout, and ensemble learning, can measure prediction confidence and highlight cases that necessitate further clinical scrutiny, rather than offering potentially deceptive high-confidence predictions. The amalgamation of XAI with calibrated probability assessments and uncertainty-aware decision support systems can greatly enhance transparency, reliability, and clinician confidence, thereby enabling the secure implementation of AI-driven biosensors for CKD screening and diagnosis.

## 8 Conclusion

Non-invasive biosensing combined with AI-driven analysis presents significant potential as an alternative to traditional serum-based diagnostics for CKD. This review highlights the notable progress in CKD diagnostics, emphasizing the transformative role of biosensors and the integration of AI. A comprehensive examination of various detection methods and sensor technologies for early CKD diagnosis has been conducted. In conclusion, saliva analysis emerges as a promising non-invasive biomarker for predicting CKD when compared to other serum-based methods. AI-enhanced non-invasive biosensors should be viewed as supplementary tools for CKD screening, ongoing monitoring, and risk stratification, rather than as direct substitutes for traditional renal function assessments. Established clinical biomarkers, such as estimated glomerular filtration rate (eGFR), serum creatinine, albuminuria, and urine albumin-to-creatinine ratio (UACR), continue to serve as the reference standards for diagnosing, staging, and managing CKD. Non-invasive biosensors provide numerous benefits, including painless sample collection, rapid POC testing, continuous monitoring, and enhanced patient compliance, making them particularly advantageous for large-scale population screenings, home monitoring, and long-term disease surveillance. Nevertheless, positive results obtained from non-invasive biosensing platforms should be interpreted in conjunction with clinical evaluations and validated through standard laboratory tests prior to making therapeutic decisions.
