# Supplementary material for: Recent advancements and future prospects on AI-integrated sensing techniques for non-invasive chronic kidney disease diagnosis: a review
Source: Front Artif Intell. 2026 Aug 13;9:1836646. doi: 10.3389/frai.2026.1836646 (PMC13518340; doi:10.3389/frai.2026.1836646)
Supplement: Supplementary file 1 [file Table_1.docx]

**Supplementary Table:** Summary of selected studies carried out for the detection of CKD using a machine learning approach

| **References** | **Dataset size** | **Input modality** | **Classification** | **Validation** | **Preprocessing** | **Feature selection** | **Model type** | **Metrics** | **Limitations** |
| --- | --- | --- | --- | --- | --- | --- | --- | --- | --- |
| (Halder et al., 2024) | 400 (250-CKD, Non-CKD-150) | Clinical data (UCI CKD Dataset) | Binary classification (CKD vs non-CKD) | Train-test validation | Missing value handling, encoding, normalization. | Correlation, Chi^2^, Variance Threshold, Recursive Feature Elimination, Sequential Forward Selection, Lasso Regression, & Ridge Regression | ML‐CKDP with RF, AdaBoost, GB, XgBoost, NB, SVM, and DT | Accuracy, Precision, Recall, F1-score, ROC-AUC | Small dataset, limited external validation |
| (Dey et al., 2022) | 400 (250-CKD, Non-CKD-150) | Clinical data (UCI CKD Dataset) | Binary classification (CKD vs non-CKD) | Train-test validation | Duplicate, cross-validation, normalization, KNN imputation technique | Chi^2^-MI hybrid strategy derived from the Wrapper feature selection method | KNN, RF, SVM, Gaussian NB, DT, Multi-Layer Perceptron (MLP), Bagging, GB, AdaBoost, CatBoost, Extra trees, LightGBM, XGBoost | 98% accuracy with Extra Trees + Chi^2^-MI | Small dataset, risk of overfitting, lack of external validation, single p ublic dataset |
| (Ghosh and Khandoker, 2024) | 491 (CKD-56, non-CKD - 435) | Clinical data values | Binary classification (CKD vs non-CKD) | 10-fold CV | refines raw data, encompassing handling missing values, scaling features, encoding variables, and addressing imbalances |  | LR, RF, DT, Naïve Bayes (NB), and extreme gradient boosting (XGBoost), XAI (SHAP and LIME) | 93.29 ± 0.041 XGBoost accuracy (%) | Biased dataset |
| (Metherall et al., 2025) | 400 (250-CKD, Non-CKD-150) | Clinical data (UCI CKD Dataset) | Binary classification (CKD vs non-CKD) | 10-fold cross-validation, | k-NN imputing, discard the 17 patients that do not have a serum creatinine, One hot encoding | AT-home, monitoring, laboratory | ANN, RF | RF achieves superior accuracy (92.5%) in at-home | Small dataset, risk of overfitting, lack of external validation, single public dataset, class imbalance risk |
| (Khalid et al., 2023) | 400 (250-CKD, Non-CKD-150) | Clinical data (UCI CKD Dataset) | Binary classification (CKD vs non-CKD) | 10-fold cross-validation | Duplicate, cross-validation | Pearson correlation method | NB, DT, RF, GNB, hybrid | Hybridmodel achieves 100 % accuracy | Small dataset, limited external validation |
| (Li et al., 2025) | 400 (250-CKD, Non-CKD-150) | Clinical data (UCI CKD Dataset) | Binary classification (CKD vs non-CKD) | 10-fold cross-validation | missing numerical values, missing nominal values | RFE, PCA | SVM, KNN, LGBM, Xg, CatBoost, Ada, hybrid, DT, RF, GB, SGB, Extra tree, ANN, HML | Hybrid model has an accuracy of 98.33 | Small dataset, limited external validation |
| (Chittora et al., 2021) | 400 (250-CKD, Non-CKD-150) | Clinical data (UCI CKD Dataset) | Binary classification (CKD vs non-CKD) | SMOTE | handle missing values, rescaling of the dataset, transform into binary data and standardize of the dataset. | Wrapper, Filter and Embedded | ANN, C5.0, LR, Linear SVM, KNN and random tree | CM, accuracy, error, precision, recall, SVM – 98.46%, DL – 99.6% | Small dataset, limited external validation |
| (Sharma et al., 2025) | 400 (250-CKD, Non-CKD-150) | Clinical data (UCI CKD Dataset) | Binary classification (CKD vs non-CKD) | k fold cross-validation | encode categorical variables, and address missing values | Correlation | ET, XgBoost, DT, GBC, SGB, CatBoost, AdaBoost, RF, KNN | XgBoost – 99.20 accurcay | Small dataset, limited external validation |
| (Gogoi and Valan, 2025) | 400 (250-CKD, Non-CKD-150) | Clinical data (UCI CKD Dataset) | Binary classification (CKD vs non-CKD) | (RFECV, 10-fold validation | categoricalvariableswere transformed into numerical formats, missing data, KNN imputation | MI, GA, BAT algorithm | RF, DT, XGBoost, XAI with SHAP | XGBoost with GA feature – 92 % accuracy | Small dataset, limited external validation |
| (Bijoy et al., 2025) | 400 (250-CKD, Non-CKD-150) | Clinical data (UCI CKD Dataset) | Binary classification (CKD vs non-CKD) | 10-fold validation | Label encoding, miss forest imputation | LASSO, fisher score, information gain, relief | LR, AdaBoost, MKR Stacking, KNN, DT, RF, MLP, MKR Voting, hyperparameter tuning, | MKR Stacking achieved the highest accuracy of 99.50% | Small dataset, limited external validation |
| (Jayashree and Anitha N, 2025) | 400 (250-CKD, Non-CKD-150) | Clinical data (UCI CKD Dataset) | Binary classification (CKD vs non-CKD)  REBEL-based | Kidney Failure Risk Equation (KFRE) | Data cleaning, normalization, missing value, handling | Quantum mutual information-based feature selection | Conceptual Metaphor Quantum Correlation + Radial Basis Extreme Learning | Accuracy: 98%, Precision: 88%, Recall: 92% | Evaluated only on benchmark dataset; lacks external clinical validation and real-world deployment analysis |
| (Sharma et al., 2026) | 500 anonymized patient records | Structured clinical data | Early CKD diagnosis | Internal validation and model comparison | Handling missing values, categorical encoding, feature normalization, train-test split | NA | Multilayer Fuzzy Inference System and Adaptive Neuro-Fuzzy Inference System | MLFIS Accuracy: 99.33%, Precision: 99.2%, Recall: 99.4%, F1-Score: 99.3%; ANFIS Accuracy: 99.5%, Precision: 99.4%, Recall: 99.6%, F1-Score: 99.5% | Broader hospital validation needed |
| (Mao et al., 2026) | GSE104948 - 170 CKD patients, 21 Living donors, GSE104954 - 169 CKD patients, 21 Living donors, GSE180395 – 91 CKD patients, 30 Living donors, GSE115857 – 79 CKD patients, 7 Living donors, GSE93798 – 20 CKD patients, 22 Living donors | Omics/gene expression + network data | Identification of CKD-related pathogenic targets | Machine learning model evaluation with SHAP interpretation | Gene screening, normalization, network construction | Machine learning feature importance | Stepwise LR + RF mode | Evaluated using F1-score and model interpretation | Primarily computational study; experimental and prospective clinical validation required |
| (Srirenga Nachiyar and Marichamy, 2026) | 400 (250-CKD, Non-CKD-150) | Clinical data (UCI CKD Dataset) | Binary classification (CKD vs non-CKD)  EGRU | Internal validation using UCI dataset | Missing value handling, normalization | Stacked Autoencoder | Enhanced Gated Recurrent Unit optimized with Improved Clouded Leopard Optimization | Accuracy: 98.75%, Precision: 98.03%, Recall: 100%, Specificity: 96.67%, F1-score: 99.01% | Small benchmark dataset; no external validation on hospital data |
| (Panse et al., 2025) | CT image dataset | Not explicitly reported | CKD diagnosis | Experimental evaluation using standard validation protocol | Data preprocessing and feature engineering | Not explicitly reported | Deep Learning classifier with Grad-CAM interpretability | AUC, Precision, Recall and F1-score (AUC reported 0.873 in comparative analysis) | Limited benchmark evaluation; external validation |
| (Nopour and Shanbehzadeh, 2026) | 1543 CKD cohort from 4 clinical centers in Tehran (Nov 2022–Dec 2024) | Demographic + clinical + laboratory variables | Five-year mortality risk prediction | Grid Search Cross-Validation | Data cleaning, imbalance, parameter optimization, missing value | univariate analyses and adjusted LR | Multiple AI/ML and Deep Learning models | Best model: F1-score 96.35%, AUROC 0.941 (95% CI 95.06–97.63) | Single-country retrospective study; prospective external validation is needed |
| **(Mehla et al., 2026)** | 400 (250-CKD, Non-CKD-150) | Clinical/tabular data (25 attributes) | Binary CKD classification | 80:20 train-test split with comparative evaluation | Missing value imputation, categorical encoding, normalization | Min-Max scaling, standard scaling, PCA | RF, XGBoost, DT, SVM, LR, KNN, Hybrid (Adaboost+RF+LR) | **RF Accuracy: 99.0%**, Precision: 98%, Recall: 99%, F1-score: 99% | Evaluated only on UCI benchmark dataset; lacks multicentre clinical validation and prospective testing |
| **(Huda et al., 2026)** | 400 (250-CKD, Non-CKD-150),  1659 CKD patients,  and  200 CKD | Structured clinical tabular data | Binary CKD prediction | 10-fold Cross Validation | Missing value imputation, data balancing using SMOTE, normalization, categorical encoding | Pearson correlation, Gain ratio, Information gain | Explainable ML framework combining RF, KNN, XGBoost, DT, Naïve Bayes, AdaBoost, ANN, LSTM, RNN, SVM and LR with SHAP/LIME | **SVM:**  **Dataset A**  **Accuracy: 100%**,  Dataset B  **Accuracy: 98.7%**,  Dataset C  **Accuracy: 100%**, | Limited to benchmark dataset; external validation remains future work |
| **(Liu et al., 2026)** | **19,068 patients** | Clinical characteristics + laboratory biomarkers | Binary CKD prediction | k-fold cross-validation strategy (k = 5) | Data cleaning, normalization, missing value processing | Biomarker importance analysis using ML algorithms | Regression-based model with GFR, LASSO, ANN, ridge regression, KNN, SVM, DT, RF | **AUC = 0.87**, | Single-region retrospective study; external validation in other high-altitude populations is required |
| **(Chen et al., 2026)** | GSE66494 (53 patients, 8 healthy controls)  GSE108112 (107 patients, 5 healthy controls)  GSE200818 (188 patients, 5 healthy controls) | m6A RNA methylation gene expression profiles | CKD risk prediction, biomarker identification, immune subtype classification | Internal ML validation with external experimental validation (qRT-PCR/immunological analysis) | Gene normalization, differential expression analysis, batch effect correction | LASSO regression, RF importance analysis | RF, LASSO, SVM and ensemble bioinformatics pipeline | **AUC: 0.91–0.97** for diagnostic signatures; robust biomarker discrimination | Based mainly on public datasets; requires large multicenter prospective clinical validation |
| **(Wang et al., 2026)** | CHARLS database (2,500) | Demographic, laboratory and clinical variables | CKD progression prediction | External cross-ethnicity validation and 5-fold cross-validation | Missing value imputation, normalization, feature scaling | Recursive Feature Elimination (RFE) | XGBoost, RF, GB ensemble, SVM, LR | **ROC-AUC: 0.89–0.93**, Accuracy ≈90%, F1-score ≈0.89 | Performance may vary across healthcare systems; prospective validation required |
| **(Yogesh et al., 2025)** | **UCI CKD dataset (400 samples)** | Clinical/tabular data (24 attributes) | Binary CKD classification | Train-test evaluation with cross-validation | Missing value imputation, normalization, categorical encoding | Hybrid Feature Selection combining filter and wrapper methods | Ensemble Deep Learning architecture | **Accuracy: 99.75%**, Precision: 99.70%, Recall: 99.80%, F1-score: 99.74%, ROC-AUC ≈0.99 | Benchmark dataset is relatively small; external multicenter validation is lacking |
| **(Kim and Woo, 2025)** | 12680 CKD cohort | Electronic health records (demographics, laboratory values, comorbidities) | Mortality prediction (death risk classification) | Internal validation using training/testing cohorts and cross-validation | Missing value handling, normalization, feature engineering | LASSO | DNN mortality prediction model | **Deep Learning - AUC: 0.83**, Accuracy 0.9683,  RF - **AUC: 0.79**, Accuracy 0.9684,  XGBoost - **AUC: 0.71**, Accuracy 0.9683, | Retrospective design; external prospective validation and explainability improvements are needed |
